# Hidden Ethnomedicinal Diversity in a Fine-Scale Study from Konak, Eastern Anatolia

**DOI:** 10.3390/plants15030383

**Published:** 2026-01-26

**Authors:** Turgay Kolaç, Narin Sadikoğlu, Mehmet Sina İçen

**Affiliations:** 1 Vocational School of Health Services, Pharmacy Services, İnönü University, Malatya 44280, Türkiye; 2 Faculty of Pharmacy, Department of Pharmacognosy, İnönü University, Malatya 44280, Türkiye; narin.sadikoglu@inonu.edu.tr (N.S.); sina.icen@inonu.edu.tr (M.S.İ.)

**Keywords:** ethnobotany, traditional medicine, medicinal plants, Malatya, Türkiye

## Abstract

This study documents the ethnomedicinal knowledge of Konak (Malatya, Eastern Anatolia, Türkiye), a region with rich plant diversity but no prior comprehensive research. The aim of the study is to systematically document and analyze the ethnomedicinal practices of Konak village, focusing on plant taxa (species, subspecies and varieties) used, preparation methods, and therapeutic applications. Data were collected through semi-structured interviews with 68 local informants. Quantitative analysis was performed using Informant Consensus Factor (F_IC_) and Use Value (UV) indices. Plant specimens were collected, identified, and deposited in the herbarium. The study documented 86 plant taxa from 35 families used in 230 therapeutic applications. Lamiaceae, Asteraceae, and Rosaceae were the most represented families. High F_IC_ values were recorded for colds (F_IC_ = 0.95), stomach pain (F_IC_ = 0.92), and inflammation (F_IC_ = 0.90), indicating strong community consensus. The most frequently cited species were *Origanum vulgare* subsp. *gracile*, *Mentha* spp., and *Rosa canina*. There are novel or locally specific uses, with 13 taxa having no previously recorded ethnomedicinal applications in the reviewed literature. The findings reveal Konak as a significant repository of ethnomedicinal knowledge. High-F_IC_ taxa represent prime candidates for phytochemical and pharmacological research to validate traditional uses and support evidence-based phytotherapy. This study enriches regional ethnopharmacological data and highlights candidate taxa for pharmacological validation.

## 1. Introduction

Plants have played a vital role in human life throughout history. Knowledge regarding their use for diverse purposes such as food, fuel, tools, and shelter has been accumulated through empirical observation and practical experience, and transmitted orally across generations to the present day [1]. Archaeological evidence including tomb wall paintings, clay tablets, and various artifacts dating back to 3000 BCE indicates that plants were among the primary resources used for disease treatment in ancient civilizations [2,3]. In early human history, disease was instinctively confronted through magical and religious practices. Over time, priest-healers emerged, combining spiritual rituals with the medicinal use of plants [1,4,5].

During the Mesopotamian civilization around 3000 BC, approximately 250 known herbal drugs were recorded [1]. In addition to invoking deities such as the Goddess of Health, the Goddess of Disease, the God of Eyes, and the God of Ears for healing, ancient societies also employed remedies derived from plants, animals, and minerals [6].

Until the 19th century, medical treatments primarily relied on herbal preparations, compound mixtures, or the direct use of plant materials. In contemporary times, traditional folk medicine remains in use, particularly in regions with limited access to modern healthcare services, with most remedies derived from plant and animal sources [3].

Globally, approximately 20,000 plant species are documented as being used for therapeutic purposes. However, when including undocumented species utilized in traditional and folk medicine, the total number is estimated to exceed 100,000. In Türkiye alone, more than 500 plant species are believed to be used for medicinal purposes [1,7,8,9,10].

The rising interest in phytotherapy is largely driven by the high cost and adverse side effects associated with synthetic drugs, as well as the lack of definitive treatments for certain diseases. In Türkiye, researchers are increasingly focusing on the country’s rich botanical diversity as a valuable resource for medicinal exploration [1]. Traditional folk remedies, transmitted through generations, have provided a foundational basis for modern pharmacological developments. In parallel, major pharmaceutical companies have renewed their interest in plant-based compounds for the discovery and development of novel therapeutics [11].

Türkiye is considered one of the richest countries in terms of plant diversity, with estimates suggesting around 12,000 plant taxa, approximately 35% of which are endemic [12,13] despite the rich ethnobotanical heritage of Türkiye, research on traditional herbal medicine remains limited. While some studies have been conducted in the Malatya region, no prior documentation exists regarding the traditional folk medicine of the Konak area [14,15,16]. Formerly known as Yukarı Banazı, Konak is one of the oldest settlements in Malatya and, despite its proximity to the city center, has retained much of its cultural identity. However, the oral transmission of traditional knowledge is increasingly at risk due to rapid sociocultural transformation. In the context of a rapidly evolving digital era, the documentation of this knowledge has become essential. By contributing these findings to the scientific literature, this study aims to enrich current understanding of traditional folk medicine and support the identification of novel phytotherapeutic agents and treatment strategies.

In line with these aims, we hypothesize that the ethnomedicinal plants used in Konak village constitute a rich and locally distinctive traditional therapeutic repertoire, characterized by high informant consensus and including several uses that have not been previously reported in the ethnobotanical literature.

## 2. Results

### 2.1. Demographic Characteristics of Study Participants

**Figure 1:**
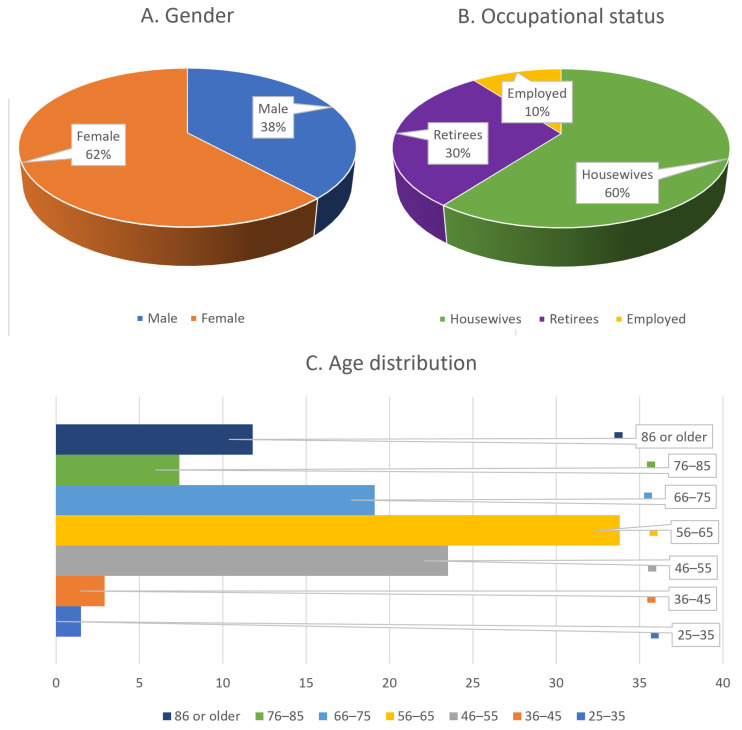
Informants’ distribution by (**A**). Gender, (**B**). Job, (**C**). Age groups (*n*: 68).

Among the 68 informants, 38.2% were male and 61.8% were female (Figure 1A). Women contributed a larger proportion of the total reported ethnobotanical knowledge (62.9%) compared to men (37.1%). However, men demonstrated a broader range of knowledge, with both the most and least knowledgeable individuals belonging to this group. While women generally exhibited higher overall familiarity with medicinal plant use, men involved in occupations that required extended exposure to natural environments—such as livestock farming, hunting, or agriculture—tended to possess more specialized and context-specific expertise.

In terms of occupational status, 60.3% of informants were housewives, 29.4% were retirees, and 10.3% were employed (Figure 1B). However, no significant correlation was observed between current occupation and the level of ethnobotanical knowledge, as informants often acquired their expertise through past professions or long-standing hobbies pursued at different stages of life.

The age distribution of participants was as follows: 1.5% were aged 25–35 years, 2.9% were 36–45 years, 23.5% were 46–55 years, 33.8% were 56–65 years, 19.1% were 66–75 years, 7.4% were 76–85 years, and 11.8% were 86 years or older (Figure 1C). Informants aged 46 to 75 years demonstrated the highest levels of ethnobotanical knowledge, while those above 75 years often faced difficulties recalling specific information. The demographic structure of the region, which is largely composed of retirees and elderly individuals, along with the intentional focus on older informants, contributed to the underrepresentation of participants younger than 45 years in the sample.

### 2.2. Plant Parts, Routes of Administration and Therapeutic Use Distribution

The most commonly utilized plant parts were the aerial parts, fruits, and leaves (Table 1). In terms of preparation and application, plants used externally were most frequently applied directly (14%), followed by use as poultices or juices after grinding (9%). For internal administration, direct consumption was the predominant method (17%), followed by processed preparations (15.5%), infusions (15.5%), and decoctions (12%). Overall, internal applications (62.4%) were reported more frequently than external ones (37%) (Table 2).

Informant Consensus Factor (F_IC_) values exceeded 0.90 in key therapeutic categories (e.g., 0.95 for colds, 0.92 for stomach pain), indicating strong communal agreement (Table 3). Species with high F_IC_ and use value (UV) should be prioritized for phytochemical and pharmacological studies.

### 2.3. Mixtures

In this study, all ethnomedicinal mixtures reported by the informants were documented comprehensively, and their preparation methods and traditional uses are presented below. These mixtures are described separately because their detailed preparation steps and culturally embedded uses could not be appropriately summarized in a table format.

Mixture-1: the root of *Arnebia decumbens* (Vent.) Coss. & Kralik used together with the gums of *Gundelia tournefortii* L. and *Pistacia terebinthus* L. To prepare the ointment, equal amounts of *G*. *tournefortii* gum and *P. terebinthus* gum are melted in butter, after which the chopped root of *A. decumbens* is added and the mixture is cooked to obtain a Vaseline-like formulation. This ointment is applied to treat hand and foot cracks and wounds.

Mixture-2: *A. decumbens* root roasted in butter is rolled into a pill and used regularly orally for stomach pains and ulcers.

Mixture-3: fresh roots of *Anchusa azurea* Mill. var. *azurea* or *Anthyllis vulneraria* L. subsp. *boissieri* (Sagorski) Bornm. roasted in butter and made into a cream and used for wound healing.

Mixture-4: fresh walnut seeds (*Juglans regia* L.) are placed on a clean plate, roasted in the embers and the soot from the smoke is collected on the plate. The same amount of fresh butter was poured over the seeds, and with the help of a toothpick, the walnut soot was collected from the plate, utilizing the butter. This collected product was applied to the eyes as kohl. Women used it for beauty purposes, and it was also used against eye diseases.

Mixture-5: sour plum (*Prunus domestica* L.) and Turkish coffee were mixed and used against diarrhea.

Mixture-6: henna is mixed with bile and applied on the wounded part of the foot. It is used externally in foot and heel cracks and wounds.

Mixture-7: olives (*Olea europaea* L.) are crushed well. The same amount of soap is added, and a dough consistency is obtained. It is tied to the broken place with a cloth.

Mixture-8: salt, dried onion (*Allium cepa* L.), raisins (*Vitis vinifera* L.) and wheat (*Triticum aestivum* L.) flour are made into a paste and wrapped in a cloth. It is used for bruises, fractures and dislocations.

Mixture-9: walnut (*J. regia*) leaves are boiled, and some henna is added in a suitable container and the feet are kept in it while the water is hot. It is used for foot fungus, odor and care.

Mixture-10: straw of aged wheat (*T. aestivum*) and *Verbascum georgicum* Benth., or *V. sphenandroides* K.Koch are boiled and cooled. Sitz baths are used for childless women and for various gynecological diseases. It is used regularly for a long time (up to 1 week). Antibacterial effect.

Mixture-11: for asthma and jaundice, *P. terebinthus* gum is dissolved in olive oil (*O. europaea*) and kept in the open air overnight. Grape juice (*V. vinifera*) is added to this mixture and used regularly.

### 2.4. Plants Used as Folk Medicine in Konak Region

Plants used as folk medicine in the Konak region are listed alphabetically by species name. These plants are tabulated with their local names, parts used, preparation techniques, usage categories, and calculated frequency of citation (Table 4). Among the 35 plant families represented by 86 taxa recorded in this study (64 genera, 68 species, 15 subspecies, and 3 varieties), the most commonly used families were Lamiaceae (14 taxa), Asteraceae (11 taxa), and Rosaceae (11 taxa).

Among the documented practices, the most widely known involved the internal use of the aerial parts of *Origanum vulgare* subsp. *gracile*, either boiled with milk or added to food, for the treatment of colds. Another highly recognized practice was the external application of the latex from *Euphorbia grisophylla* and *E. macrocarpa* for treating bee, scorpion, and snake bites. These applications were known to 69% of informants (Table 4).

The second most widely cited practices included the internal use of infusions prepared from the aerial parts of *Pelargonium endlicherianum*, and the consumption (as a decoction or jam) of *Rosa canina* fruits for managing common colds, reported by 68% of participants (Table 4).

The third most common practices involved the internal consumption of infusions made from the aerial parts of *Mentha longifolia* subsp. *longifolia*, *M. pulegium*, and *M. spicata* subsp. *spicata*. These were used for the treatment of colds, nausea, stomach pain, and inflammation, either by boiling with milk or incorporating into meals. These uses were cited by 66% of informants (Table 4).

Some of the least commonly reported ethnomedicinal practices were known only to a small number of individuals, with corresponding plant species recognized exclusively by those informants. These rare applications, cited by approximately 1.5% of participants, include the following examples (Table 4):

The bulbs of *Allium ampeloprasum* are roasted over charcoal, and the resulting material is applied externally to treat inflammation and injuries caused by glass or thorns. Additionally, the juice extracted from crushed bulbs is used for bee and scorpion stings. An infusion prepared from the aerial parts of *Cyclotrichium niveum*, or its internal consumption by adding it to food, has been reported for treating colds, nausea, stomach pain, and inflammation. *Pulicaria dysenterica* subsp. *dysenterica* is used both internally and externally: its infusion or direct consumption is reported for internal inflammation, while a paste prepared from the plant is applied for external inflammation. Leaves of *Salvia euphratica* var. *euphratica* and *S. multicaulis* are used in the form of infusions for managing eczema and pruritic (itchy) skin conditions.

## 3. Discussion

Konak is one of the oldest settlements in Malatya and remains among the few regions where no comprehensive ethnobotanical assessment has previously been conducted—largely due to its historically closed cultural structure. During the fieldwork, the urgency of documenting traditional knowledge became increasingly evident, as the passing of several informants resulted in the irreversible loss of valuable ethnomedicinal information. This highlights the critical need to preserve such knowledge through systematic documentation in digital and scientific archives and to ensure its timely publication. In the context of Anatolia’s rapidly eroding biocultural heritage, the significance of localized studies such as this continues to grow.

Field observations indicated that while certain plants are still in use, their local names or methods of application are often forgotten. Herbarium specimens were collected for unidentified taxa; however, due to uncertainties in nomenclature and usage, these species were excluded from the current analysis. These underscores emerging challenges in ethnobotanical cataloging and points to the need for future studies focused on local naming conventions and oral taxonomy.

Participant demographics—participants were primarily in the 46–75 age group—suggest a waning interest among younger generations in traditional knowledge systems. This trend, coupled with the community’s close proximity to the city center (3 km), which facilitates access to modern medical services, contributes to the observable decline in folk medicinal practices. The underrepresentation of individuals under 45 years of age further limits the generalizability of the findings to the broader population. Although rigorous measures were taken to exclude data influenced by modern media sources, thereby enhancing the reliability of the information collected, further research is needed to explore how this traditional knowledge may be integrated with contemporary medical frameworks in a culturally sensitive and scientifically validated manner.

An analysis of the number of remedies reported across different age groups revealed that the highest counts were generally provided by individuals aged 76 years and older. However, this trend was challenged by two notable exceptions: a 60-year-old and a 52-year-old male informant were among those with the most extensive ethnobotanical knowledge. These cases suggest that, while traditional knowledge is predominantly concentrated among the elderly, younger individuals—particularly men—can also serve as significant repositories of such information. Their extensive knowledge was attributed to personal curiosity and lifestyle factors, such as spending prolonged periods in remote mountainous regions for pastoral activities or hunting.

The use of semi-structured interviews in this study, rather than standardized questionnaires, was a deliberate methodological choice aimed at capturing the multidimensional, culturally embedded, and experiential nature of folk medicine traditions. Closed-ended questionnaires, while useful for quantitative analysis, often constrain responses within predefined categories, thereby limiting the opportunity to uncover unanticipated or nuanced information. In contrast, semi-structured interviews offered the flexibility to follow the natural flow of participants’ narratives, probe responses in greater depth, and elicit rich qualitative data—including details on knowledge transmission, preparation techniques, and context-specific practices. This approach allowed for a more authentic representation of the narrative and situational character of traditional medical knowledge, thereby enhancing both the validity and the depth of the collected data in this study, the most preferred plant parts were aerial parts, leaves, and fruits (Table 1), aligning with patterns in other Turkish ethnobotanical research. Similarly, plants recommended for stomach and respiratory disorders mirrored those in Malatya and neighboring provinces [14,15,17,18,19,20,21,22,23,24].

Konak (Malatya, Türkiye) represents a unique ethnobotanical site due to its rich plant diversity and insular cultural dynamics. This study documented 86 distinct plant taxa(species, subspecies and varieties) used in 230 traditional remedies, with high Informant Consensus Factor (F_IC_) values underscoring the reliability and potential pharmacological significance of local knowledge. Of these taxa, 60 species were found to be native (wild) to the region. however, for *Vitis vinifera* L., the variety known as “Banazı Karası” is unique to this area, representing a locally adapted cultivar with significant cultural and genetic value. Notably, taxa such as *Origanum vulgare* subsp. *gracile*, *Mentha* species, and *Rosa canina* exhibited high Use Values (UV: 0.66–0.69), reflecting their widespread and consistent therapeutic use within the community.

In addition to high-frequency applications, several specialized uses emerged. The powdered aerial parts of *Ajuga chamaepitys* subsp. *chia* and *A. chamaepitys* subsp. *laevigata* were reported to treat deep burns effectively, allegedly without leaving scars. Similarly, while daisy species (*Bellis* spp.) were frequently used as antiseptics for open wounds, *Tripleurospermum oreades* was reported to be even more effective. Given its documented antitussive activity suggesting airway-relaxing effects similar to β2-agonists [25], *Astragalus gummifer*’s use for respiratory disorders merits further phytochemical and pharmacological studies. *Plantago lanceolata* (and possibly *P. major* subsp. *intermedia*) was regarded as highly effective for various ailments, while *Polygonum patulum* was cited as particularly suitable for treating eczema. Of the polyherbal treatments, mixtures labeled 1 and 3 were especially noteworthy and merit further study. Both mixtures were highly regarded by the majority of experts and were recommended for further consideration, with particular emphasis placed on their potential for development into commercially viable products derived from natural sources.

Although the use value (UV) of *Crataegus orientalis* subsp. *orientalis* for cardiovascular conditions is low, informants who reported this use stated that they obtained notably positive results from the plant. This strong individual-level perception has led to limited local trade of the species. Its lack of widespread popularity within the region may be related to inadequate marketing or the deliberate concealment of the plant’s identity by those involved in its trade. Moreover, although the use of the root has been rarely addressed in the scientific literature, informants emphasized that the root is more effective than other plant parts (Appendix A).

The most widely known remedies (UV: 0.69) included the internal consumption of *Origanum vulgare* subsp. *gracile* aerial parts—typically boiled in milk or added to food—to treat colds, and the external application of latex from *Euphorbia grisophylla* and *E. macrocarpa* for insect and animal bites. Interestingly, some informants also reported the use of these latexes for eye infections. However, these applications were described as harmful, with reports of severe adverse effects including vision loss. Consequently, these practices were excluded from the catalog of medicinal uses, yet they highlight the critical need for awareness of potentially toxic traditional remedies.

The second most commonly cited remedies (UV: 0.68) involved infusions of *Pelargonium endlicherianum* and fruit decoctions or jam from *Rosa canina*, used internally to treat colds. Third in prevalence (UV: 0.66) were infusions of aerial parts from *Mentha longifolia* subsp. *longifolia*, *M. pulegium*, and *M. spicata* subsp. *spicata*, used to manage colds, nausea, stomach pain, and inflammation—either by boiling in milk or incorporating into meals.

Conversely, some of the least known remedies (UV: 0.02) included the use of roasted bulbs of *Allium ampeloprasum* for inflammation and thorn injuries or juiced for stings; infusions or culinary use of *Cyclotrichium niveum* for cold-related symptoms; and *Pulicaria dysenterica* subsp. *dysenterica* used internally for inflammation and externally as a paste. Infusions prepared from the leaves of *Salvia euphratica* var. *euphratica* and *S. multicaulis* were reported for the treatment of eczema and itchy skin conditions.

Interestingly, even low-use-value plants such as *Cornus mas* were embedded in local oral traditions and folk narratives-such as tales of reviving the dying-suggesting symbolic or historical importance. This underlines the necessity of integrating quantitative measures (e.g., UV, F_IC_) with qualitative cultural insights, particularly for conditions that may have been historically undiagnosable, such as cardiovascular disease.

Approximately 49% of the documented plant uses in the Konak region (42 out of 86 taxa) have not been previously recorded in ethnobotanical studies from surrounding areas [18,19,20,21,22,23,24,26]. Moreover, 95% of the commonly cited species are employed for different purposes or prepared using distinct methods compared to their use in nearby regions. For instance, in Konak, *Rosa canina* is used to treat colds in the form of jam or decoction, whereas in Kürecik, its use is limited to decoction alone. Such differences in usage patterns may be attributed to Konak’s relative geographical isolation, region-specific culinary traditions, and varying degrees of access to plant resources [14,15,17,18,19,20,21,22,23,24].

By comparison, a previous province-wide ethnobotanical survey of Malatya documented 108 taxa from 39 families, with *Asteraceae* (21 taxa), *Lamiaceae* (14 taxa), and *Rosaceae* (13 taxa) also being the most frequently cited families [15]. Notably, our study recorded 8 families, 29 genera, and 56 species not included in the provincial survey. Of the 30 overlapping species, 18 were found to have entirely different uses or preparation methods in the Konak region.

Taxa with consistent uses across both datasets include *Anchusa azurea* var. *azurea*, *Bellis perennis*, *Cyclotrichium niveum*, *Cydonia oblonga*, *Mentha spicata* subsp. *spicata*, *Pelargonium endlicherianum*, *Plantago lanceolata*, *Prunus armeniaca*, *Quercus infectoria* subsp. *infectoria*, *Rosa canina*, *Teucrium polium* subsp. *polium*, *Urtica dioica* subsp. *dioica*, and *Vitis vinifera* [14,15].

When compared with the broader dataset from Malatya, 65% of the taxa reported in our study differed either in species or genus [15]. The primary reason for this substantial divergence is that the Konak region was not included in the scope of the earlier survey. This highlights the importance of conducting ethnobotanical research at a finer geographical scale, as broader regional studies may overlook localized knowledge systems. Preserving such localized ethnomedicinal traditions is essential for safeguarding biocultural diversity and advancing future pharmacological research.

In comparison to the study conducted in the neighboring province of Elazığ, our study recorded 11 families, 35 genera, and 56 species that were not included in that survey. Although there were 30 species common to both studies, 20 of these were employed for entirely different purposes and preparation methods. The taxa with similar uses were identified as *Allium sativum*, *Bellis perennis*, *Anchusa azurea* var. *azurea*, *Elaeagnus angustifolia*, *Mentha longifolia* subsp. *longifolia*, *M. spicata* subsp. *spicata*, *Malva neglecta*, *Rosa canina*, *Urtica dioica* subsp. *dioica*, and *Vitis vinifera* [18].

Regarding the study conducted in the Andırın district of another neighboring province, Kahramanmaraş, our study documented 14 families, 43 genera, and 74 species that were not present in that report. While there were 11 shared species, 10 were utilized for completely different purposes and preparation methods. The taxa displaying similar uses are *Bellis perennis* and *Tragopogon dubius* subsp. *dubius* [21].

Ethnobotanical studies contribute to the scientific literature not only by validating previously reported plant uses but also by identifying novel applications. In countries such as Türkiye, where the endemism rate exceeds 35%, the documentation of previously unreported uses is both expected and significant. In the present study, 13 of the 86 recorded taxa—*Anthemis haussknechtii*, *Anthyllis vulneraria* subsp. *boissieri*, *Arnebia decumbens*, *Crocus damascenus*, *Echinops pungens*, *Eminium intortum*, *Galium spurium* subsp. *ibicinum*, *Geranium tuberosum*, *Polygonum patulum*, *Salvia euphratica* var. *euphratica*, *Verbascum georgicum*, *V. sphenandroides*, and *Vicia hybrida*—were found to have no previously documented ethnobotanical uses, representing their first appearance in the literature. Although these taxa are not endemic, their limited regional distribution may account for the lack of prior records (Appendix A).

These findings highlight both the originality of the present study and the potential for uncovering additional traditional uses through similar surveys in neighboring areas. A substantial portion of the documented remedies align with previously published data, affirming cultural continuity. At the same time, numerous novels or off-label applications significantly expand the existing body of ethnomedicinal knowledge.

Species with high Informant Consensus Factor (F_IC_) values and citation frequency-particularly those clustered in categories of widespread or consistent use-emerge as strong candidates for further phytochemical and pharmacological investigation. Some of these plants may hold promise for future clinical or preclinical studies. Overall, the results demonstrate that the Konak region harbors a distinct and valuable repository of traditional medical knowledge, with significant potential to enrich ethnobotanical literature and inform drug discovery efforts.

## 4. Materials and Methods

### 4.1. Study Area

Malatya, situated in the Upper Euphrates region of Eastern Anatolia, is located between 35°54′ and 39°03′ north latitudes and 38°45′ and 39°08′ east longitudes. Granted metropolitan status on 12 November 2012, it is the largest city in the region, encompassing a total area of 12,313 km^2^. The province lies at an elevation of 977 m above sea level and, as of 2016, had a population of 781,305, distributed across 13 districts. Malatya experiences a continental climate characterized by cold, harsh winters; however, recent climatic changes associated with global warming have led to milder seasonal conditions [15,27,28]. The name “Malatya” is derived from the Hittite word “Melid,” meaning honey [29]. Settlement areas believed to have been established during the Byzantine period include Gündüzbey, Yeşilyurt, Yakınca, Banazı, Bostanbaşı, and Tecde [30]. Banazı, one of the oldest settlements, was later renamed “Konak” [31].

**Figure 2:**
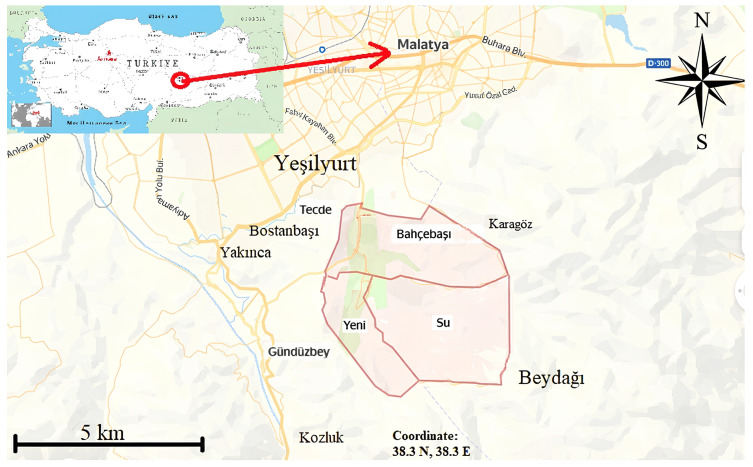
Neighborhoods of Konak: Su, Bahçebaşı, and Yeni.

Situated on the southern slopes of Beydağı, Konak functioned as an independent township until 2012. Following Malatya’s designation as a metropolitan municipality, Konak—located just 3 km from the city center—was incorporated into the Yeşilyurt district. The settlement currently comprises three neighborhoods: Su, Bahçebaşı, and Yeni (Figure 2). Despite its proximity to the urban center, Konak has retained much of its cultural heritage, more so than many remote villages. The local population is recognized for its hospitality, although permanent settlement by outsiders is generally discouraged. According to oral histories, the village was originally founded by seven families, and the present population of approximately 7000 is largely descended from these early settlers [27,31,32]. Studying such a culturally rich settlement will make a significant contribution to the literature.

### 4.2. Interviews with Local People

Although over 100 individuals were interviewed during the fieldwork, ethnobotanical data were successfully obtained from 68 informants. The informants were specifically selected from among individuals renowned within the community for their extensive knowledge of plants. A total of 243 herbarium specimens were collected based on information provided by these participants. For each plant, detailed records were made, including local names, plant parts used, preparation methods, medicinal applications, collection sites, collection periods, and modes of application.

Plant specimens were collected with the assistance of local informants, photographed in situ, and subsequently archived. These archived images were later utilized to aid in follow-up interviews. Participants were asked to share information about folk remedies they had personally used, observed, or heard of through oral tradition, explicitly excluding knowledge acquired via modern communication sources such as television, newspapers, or the internet. Data collection was conducted through semi-structured interviews rather than standardized questionnaires to allow for greater flexibility and depth. Semi-structured interviews are a scheduled qualitative data collection method based on open-ended questions that follow a general interview guide and cover a predefined set of topics [33]. Group interviews involving two to three participants were found to be particularly effective, as they facilitated the confirmation of shared knowledge and encouraged participants to acknowledge unfamiliar topics more openly.

Nearly all informants emphasized that the practices they reported were either personally experienced, observed within their family, or heard from trusted community sources. To ensure data reliability, only those uses that had been practiced or directly witnessed were included in the study; unverifiable claims or hearsay were excluded. Given the prevalence of misinformation in the modern information landscape, particular care was taken to document only widely recognized and locally validated traditional uses.

### 4.3. Plant Materials

Plant specimens were collected from Konak and its surrounding areas between May 2015 and November 2016. Specimens were carefully selected to ensure ease of taxonomic identification. Standard field equipment—including shovels, gloves, garden shears, knives, collection bags, newspapers, drying papers, plant presses, cardboard, and thread—was used to facilitate proper handling and drying of the collected materials [34]. To prevent insect infestation, the dried specimens were stored at −25 °C for three days before being mounted on herbarium sheets. The collected specimens were deposited in the herbarium of the Faculty of Pharmacy at İnönü University. “Voucher No” are provided in Appendix A.

Plant species were identified using “Flora of Türkiye and the East Aegean Islands” [35]. Scientific names were updated according to “The List of Turkish Plants—Vascular Plants” [12]. Coordinates and altitudes were recorded and verified using Google Earth and mobile GPS devices. Photographs were taken using a Canon EOS 700D camera with an 18–55 mm lens.

In this study, ethnobotanical knowledge was defined based on both the number of plant taxa reported by each informant and the total number of distinct medicinal uses they described. Each use-report was counted independently, regardless of whether multiple uses were associated with the same plant taxon. This dual-parameter approach allowed us to quantify the breadth (number of taxa) and depth (number of use-reports) of individual ethnobotanical knowledge.

### 4.4. Calculations

IBM SPSS V24 was used for graphical representation and statistical analysis. The informant consensus factor (F_IC_) was utilized in the calculations. The formula used for the calculation is: F_IC_ = (N_ur_ − N_t_)/(N_ur_ − 1). N_ur_ is the number of use-reports per category; N_t_ is the number of taxa used for that category (Table 3). This method evaluates the homogeneity of the information. F_IC_ values are low (close to 0) if plants are chosen randomly or if informants do not share knowledge about their uses, whereas values are high (close to 1) when there is a well-defined selection criterion in the community and/or knowledge is transmitted among informants. Previous studies have used “F_IC_” and “ICF” interchangeably for this factor [36,37,38,39,40,41].

The percentage of taxa was calculated using the following formula: Number of taxa in therapeutic effects/Total number of taxa × 100 (Table 3).

The percentage of species was calculated as: Number of species in the category/Total number of plant species × 100 (Table 3).

The number and percentage of plant parts used were determined (Table 1).

Calculations were also performed for the administration methods of taxa (Table 2). Percentage results were obtained using: Number of application methods in the category/Total number of application methods × 100 [37,38,39,40,41,42].

The use value (UV), a quantitative measure indicating the relative importance of taxa in local knowledge, was calculated using the formula: UV = U/N, where UV denotes the use value of a taxa, U represents the number of citations per taxa, and N indicates the number of informants [38].

## 5. Conclusions

This study provides the first fine-scale ethnomedicinal assessment of Konak (Malatya, Türkiye), offering clear support for the hypothesis presented in the Introduction: that the village maintains a rich and locally distinctive therapeutic repertoire shaped by strong informant consensus and several previously undocumented plants use. The documentation of 86 plant taxa across 230 medicinal applications confirms that Konak remains an important repository of biocultural heritage within Eastern Anatolia. High F_IC_ values for major ailment categories and the prominence of widely cited species such as *Origanum vulgare* subsp. *gracile*, *Mentha* spp., and *Rosa canina* demonstrate both the reliability and the internal coherence of local knowledge systems.

## Figures and Tables

**Table 1:** Percentage distribution of plant parts used.

Parts Used	Percentage
Aerial part	19.9
Fruit and part (fruit stalk, fruit peel, etc.)	16.5
Leaf	15.1
Flower, bud and capitulum	13.2
Root, tuber, bulb, tuber, etc. below ground	12.3
Seed	9.9
Whole plant	5.6
Stem, branch and bark	3.3
Other (Receptacle, spathe, latex, etc.)	4.2

**Table 2:** Routes of administrations and percentages.

Route of Administration	Percentage
External uses	37.6
Directly	14.1
Grind and poultice or juice	9.2
Processed via various methods	4
Infusion	4.1
Decoction	2.5
Ointment	2.1
Others	1.6
Internal uses	62.4
Directly	17
Processed via various methods	15.5
Infusion	15.5
Decoction	12
Grind and poultice or juice	2
Others	0.4

**Table 3:** Usage rates according to therapeutic effects.

Therapeutic Effects	n_t_	n_ur_	F_IC_
Mouth sores and aphthae	1	4	1
Pain relief	10	47	0.8
Allergy and allergic skin reactions	2	10	0.89
Scorpion and bee stings	7	121	0.95
Asthma and dyspnea	13	57	0.79
Foot fungus and odor	2	6	0.8
Against intestinal parasites	5	51	0.92
Nosebleeds	6	17	0.69
Boils	2	11	0.9
Diabetes	1	2	1
Toothache and inflammation	5	16	0.73
Eczema	3	5	0.5
Joint and rheumatism pain	3	66	0.97
Hand and foot cracks and care	6	29	0.82
Eye diseases	7	22	0.71
Goiter and other thyroid diseases	1	2	1
Animal diseases	4	9	0.63
External and internal infections	12	106	0.9
Indigestion	2	8	0.86
Hemorrhoids	6	16	0.67
Urinary tract diseases	5	10	0.56
Diarrhea stopper	6	23	0.77
Unwanted pregnancy	3	7	0.67
Constipation	1	2	1
Gynecology and infertility treatment	5	17	0.75
Cardiovascular diseases	4	10	0.67
Fractures, dislocations and contusions	5	47	0.91
Cosmetics	7	35	0.82
Stomach pain and ulcers	21	254	0.92
Nausea	9	169	0.95
Calluses, warts	1	3	1
Cough and phlegm	5	26	0.84
Scalp diseases and scabies	2	7	0.83
Jaundice	3	6	0.6
Smoking and alcohol addiction	1	3	1
Colds	21	361	0.94
Heel spurs	1	3	1
Strengthening the immune system	10	51	0.82
Wounds and burns	13	47	0.74
High blood pressure	2	7	0.83
Other purposes	7	22	0.71
Total	230	1715	33.9

n_t_ = Number of Taxa, n_ur_ = number of mentions in each usage category, F_IC_ = Informant Consensus Factor.

**Table 4:** Medicinal plants of Konak.

Botanical Name (Family)	Local Name/N **	Part Used	Method of Preparation	Usage Categories	UV *
*Ajuga chamaepitys* subsp. *chia* (Schreb.) Arcang. (Lamiaceae)	-/N	Whole plant	Powder eaten raw	Stomachaches and inflammations	0.03
			Infusion used orally		
			Powder eaten raw	Asthma and dyspnea	
			Infusion used orally		
			By sprinkling powder	Wounds and burns	
*Ajuga chamaepitys* subsp. *laevigata* (Boiss.) P.H.Davis (Lamiaceae)	-/N	Whole plant	Powder eaten raw	Stomachaches and inflammations	0.03
			Infusion used orally		
			Powder eaten raw	Asthma and dyspnea	
			Infusion used orally		
			By sprinkling powder	Wounds and burns	
*Alcea apterocarpa* (Fenzl) Boiss. (Malvaceae)	Horoz gülü, ibibik gülü/N	Flower	Infusion used orally	Antitussive	0.09
		Root	By sprinkling powder	Hair care	
*Allium ampeloprasum* L. (Amaryllidaceae)	Dağ soğanı/N	Bulb, Leaf	Roasted and fixed externally	Topical anti-inflammatory	0.02
				Embedded foreign bodies	
			Fresh juice is dripped externally	Venomous stings	
			Eaten raw	Systemic inflammation	
*Allium cepa* L. (Amaryllidaceae)	Soğan	Bulb, Leaf	Roasted and fixed externally	Topical anti-inflammatory	0.19
				Embedded foreign bodies	
			Fresh juice is dripped externally	Venomous stings	
			Fresh juice is mixed with honey and used orally	Tuberculosis and pulmonary diseases	
			Mixture-8	Traumatic injuries	
		Onion peel	Decoction used orally	Abortive	
*Allium sativum* L. (Amaryllidaceae)	Sarımsak	Bulblet	Swallowed	Antihypertensive	0.06
*Anchusa azurea* Mill. var. *azurea* (Boraginaceae)	Sığır emeceği/N	Root	Mixture-3	Wounds and burns	0.04
*Anethum graveolens* L. (Apiaceae)	Dereotu, samıt, tere out/N	Aerial part	Boiled and eaten	Topical anti-inflammatory	0.06
			Eaten raw	Weight loss	
*Anthemis candidissima* Willd. ex Spreng. (Asteraceae)	Papatya/N	Capitulum	Dry powder applied externally	Antiseptic	0.19
			Dry powder administered nasally	Epistaxis	
				Rhinorrhea	
				Ocular diseases	
			Infusion used orally	Common cold	
				Stomachache	
			Drink boiled with milk	Common cold	
				Stomachache	
*Anthemis haussknechtii* Boiss. & Reut. (Asteraceae)	Papatya/N	Capitulum	Dry powder applied externally	Antiseptic	0.19
			Dry powder administered nasally	Epistaxis	
				Rhinorrhea	
				Ocular diseases	
			Infusion used orally	Common cold	
				Stomachache	
			Drink boiled with milk	Common cold	
				Stomachache	
*Anthyllis vulneraria* L. subsp. *boissieri* (Sagorski) Bornm. (Fabaceae)	Emecek otu/N	Root	Mixture-3	Wounds and burns	0.05
*Arnebia decumbens* (Vent.) Coss. & Kralik (Boraginaceae)	Kızıl enik kökü/N	Root	Mixture-1	Skin conditions	0.10
			Mixture-2	Stomachache and ulcer	
			External decoction	Antiseptic for wounds	
			Mixture-1	Insect bites	
			Mixture-1	For veterinary use	
			External decoction	Insect bites	
*Astragalus gummifer* Labill. (Fabaceae)	Geven, keven/N	Aerial part	Gel from inside the woody structure-Infusion used orally	Asthma and dyspnea	0.07
				Analgesic	
		Root	Decoction used orally	For veterinary use	
				Aphrodisiac	
*Bellis perennis* L. (Asteraceae)	Papatya/N	Capitulum	Dry powder applied externally	Antiseptic	0.19
			Dry powder administered nasally	Epistaxis	
				Rhinorrhea	
				Ocular diseases	
			Infusion used orally	Common cold	
				Stomachache	
			Drink boiled with milk	Common cold	
				Stomachache	
*Brassica oleracea* L. (Brassicaceae)	Lahana	Leaf	Infusion used orally	Urological disorders and nephrolithiasis	0.03
*Capsicum annuum* L. (Solanaceae)	Acı biber	Fruit	Eaten raw	Anthelmintic	0.05
			Externally with honey	Heel spur	
*Cicer arietinum* L. (Fabaceae)	Nohut	Seed	Powder externally	Toothache	0.02
			Poultice externally	Toothache	
*Citrus limon* (L.) Osbeck (Rutaceae)	Limon	Fruit juice	Mixed with yogurt	Hypertension	0.05
*Cornus mas* L. (Cornaceae)	Kızılcık	Fruits	Drinking as a sherbet	Anti-aging	0.05
*Crataegus monogyna* Jacq. (Rosaceae)	Yemişen/N	Fruits	Eaten raw	Immunostimulant	0.07
*Crataegus orientalis* Pall. ex M.Bieb. subsp. *orientalis* (Rosaceae)	Alıç/N	Flowers	Infusion used orally	Immunostimulant	0.09
				Expectorant	
		Fruits	Eaten raw	Immunostimulant	
		Roots	Decoction used orally	Infertility	
				Cardiovascular health	
*Crocus damascenus* Herb. (Iridaceae)	Çiğdem/N	Bulb	by boiling with milk	Common cold	0.09
*Cucumis sativus* L. (Cucurbitaceae)	Salatalık, hıyar	Fruit, Cortex	Topical application of slices	Allergic skin diseases	0.05
				Burns	
*Cyclotrichium niveum* (Boiss.) Manden & Scheng (Lamiaceae)	Erzurum nanesi/N	Aerial part	Infusion used orally	Stomachaches and inflammations	0.02
				Nausea	
				Common cold	
			Eaten raw	Stomachaches and inflammations	
				Nausea	
				Common cold	
*Cydonia oblonga* Mill. (Rosaceae)	Ayva	Leaf	Infusion used orally	Analgesic	0.07
				Common cold	
		Seed	Decoction used orally	Analgesic	
				Common cold	
		Fruit	Drinking as a Compote	Stomachaches and inflammations	
				Analgesic	
*Echinops pungens* Trautv. (Asteraceae)	Deve dikeni, topuz dikeni/N	Receptacle	Decoction used orally	Anti-aging	0.03
			Decoction is applied externally	Skin tonic	
				Analgesic	
*Elaeagnus angustifolia* L. (Elaeagnaceae)	İğde/N	Fruit	Eaten raw	Common cold	0.05
*Eminium intortum* (Banks & Sol.) Kuntze (Araceae)	Susam out/N	Spathe	Drink the water in spathe	Antidipsic	0.02
*Euphorbia grisophylla* M.L.S.Khan. (Euphorbiaceae)	Sütleğen/N	Latex	Dermal drops	Pain management for venomous injuries	0.69
*Euphorbia macrocarpa* Bioss. & Buhse. (Euphorbiaceae)	Sütleğen/N	Latex	Dermal drops	Pain management for venomous injuries	0.69
*Ficus carica* L. subsp. *rupestris* (Hausskn.) Browicz (Moraceae)	İncir/N	Latex	Dermal drops	Antiseptic for wounds	0.05
				Venomous stings	
				Corn-wart treatment	
*Galium spurium* L. subsp. *ibicinum* (Boiss. & Hausskn.) Ehrend. (Rubiaceae)	Kardaş kınası/N	Aerial part	Topical henna	Skin conditions	0.07
*Geranium tuberosum* L. (Geraniaceae)	Tömlük, kömlük/N	Tuber	Soup preparation	Systemic inflammation	0.05
*Glycyrrhiza glabra* L. (Fabaceae)	Biyam, meyan	Root	Decoction used orally	Asthma and dyspnea	0.02
				Common cold	
				Tonic	
*Gundelia tournefortii* L. (Asteraceae)	Kenger/N	Seed	Decoction used orally	Common cold	0.10
				Asthma and dyspnea	
		Root	Gum placed in the nose	Epistaxis	
			Mixture-1	Skin conditions	
				Venomous stings	
*Helichrysum arenarium* (L.) Moench (Asteraceae)	Altın otu, ölmez otu/N	Capitulum	Dry powder administered nasally	Rhinorrhea	0.05
*Helichrysum plicatum* DC. subsp. *plicatum* (Asteraceae)	Altın otu, Ölmez otu/N	Capitulum	Dry powder administered nasally	Rhinorrhea	0.05
*Hordeum vulgare* L. (Poaceae)	Arpa	Seed	Dough applied topically	Traumatic injuries	0.06
				Topical anti-inflammatory	
			Poultice applied topically	Wounds and burns	
			Flour is used	Nausea	
*Juglans regia* L. (Juglandaceae)	Ceviz	Seed pericarp	Juice and poultice applied topically	Venomous stings	0.12
				Tinea pedis	
				Scalp dermatoses	
		Seed	Swallowed unripe	Ocular diseases	
				Stomachaches and inflammations	
			Infusion from the fibrous structure used orally	Analgesic	
				Vertigo	
				Systemic inflammation	
			Eaten raw	Anthelmintic	
			Mixture-4	Ocular diseases	
		Leaf	Juice and poultice applied topically	Venomous stings	
				Tinea pedis	
				Scalp dermatoses	
			Mixture-9	Tinea pedis	
		Flower	Poultice applied topically	Analgesic	
*Malus domestica* (Suckow) Borkh. (Rosaceae)	Elma	Fruit	Topical henna from peel	Skin conditions	0.07
			Eaten raw	Analgesic	
			Decoction used orally	Antitussive	
		Leaf	Topical henna	Skin conditions	
			Infusion or eaten raw	Analgesic	
*Malva neglecta* Wallr. (Malvaceae)	Ebegümeci, ebemgümeci/N	Aerial part	Infusion used orally	Systemic inflammation	0.09
			Cooked with food and eaten	Systemic inflammation	
			Sitz bath with infusion	Hemorrhoids	
			Infusion used orally	Abortive	
			By sprinkling powder	Wounds and burns	
			Juice used externally	Wounds and burns	
*Malva sylvestris* L. (Malvaceae)	Ebegümeci, ebemgümeci/N	Aerial part	Infusion used orally	Systemic inflammation	0.09
			Cooked with food and eaten	Systemic inflammation	
			Sitz bath with infusion	Hemorrhoids	
			Infusion used orally	Abortive	
			By sprinkling powder	Wounds and burns	
			Juice used externally	Wounds and burns	
*Mentha longifolia* (L.) L. subsp. *longifolia* (Lamiaceae)	Nane, nerpiz, yarpuz, dağ nanesi/N	Aerial part	Infusion used orally	Stomachaches and inflammations	0.67
				Nausea	
				Common cold	
			Eaten raw	Stomachaches and inflammations	
				Nausea	
				Common cold	
			Drink boiled with milk	Gastritis	
*Mentha pulegium* L. (Lamiaceae)	Nane, nerpiz, yarpuz, dağ nanesi/N	Aerial part	Infusion used orally	Stomachaches and inflammations	0.67
				Nausea	
				Common cold	
			Eaten raw	Stomachaches and inflammations	
				Nausea	
				Common cold	
			Drink boiled with milk	Gastritis	
*Mentha spicata* L. subsp. *spicata* (Lamiaceae)	Nane, nerpiz, yarpuz, dağ nanesi/N	Aerial part	Infusion used orally	Stomachaches and inflammations	0.67
				Nausea	
				Common cold	
			Eaten raw	Stomachaches and inflammations	
				Nausea	
				Common cold	
			Drink boiled with milk	Gastritis	
*Morus alba* L. (Moraceae)	Dut	Fruit	Its molasses is mixed with butter and consumed orally	Common cold	0.34
				Asthma and dyspnea	
				Stomachaches and inflammations	
				Stomachaches and inflammations	
			Its molasses is consumed orally	Anemia and avitaminose	
			Its molasses is applied to the eye	Ocular diseases	
			Fruit leather is applied topically	Abscesses	
				Sore throat	
			Dry fruits is applied topically	Abscesses	
		Leaf	Eaten raw	Analgesic	
			Infusion is applied topically	Skin tonic	
		Cortex	Decoction is applied topically	Venomous stings	
*Morus nigra* L. (Moraceae)	Karadut	Fruit	Juice dermal drops	Antiseptic for wounds	0.06
			Eaten raw	Aphthae	
			Juice dermal drops	Aphthae	
			Gargling with molasses	Aphthae	
			Eaten raw	Anti-aging	
*Olea europaea* L. (Oleaceae)	Zeytin	Fruit	Mixture-7	Traumatic injuries	0.03
			Mixture-11	Asthma and dyspnea	
				Jaundice	
*Origanum vulgare* L. subsp. *gracile* (K.Koch) Ietsw. (Lamiaceae)	Anıh/N	Aerial part	Drink boiled with milk	Stomachaches and inflammations	0.69
				Nausea	
				Common cold	
			Infusion used orally	Common cold	
*Pelargonium endlicherianum* Fenzl (Geraniaceae)	Solucan out/N	Aerial part	Infusion used orally	Anthelmintic	0.68
*Petroselinum crispum* (Mill.) Fuss (Apiaceae)	Maydanoz	Aerial part	Boiled and eaten	Topical anti-inflammatory	0.02
*Pistacia terebinthus* L. (Anacardiaceae)	Menengiç	Latex	Mixture-1	Skin conditions	0.05
				Insect bites	
			Placed in the nose	Epistaxis	
			Mixture-11	Asthma and dyspnea	
				Jaundice	
*Plantago lanceolata* L. (Plantaginaceae)	Damarlı yaprak, hava yaprağı, Damarlı ot/N	Seed	Eaten raw	Headache	0.41
			Decoction used orally	Antidiarrheal	
		Leaf	Chews	Toothache	
			Eaten as a salad	Thyroid disorders	
			Topical application of slices or whole	Analgesic and anti-inflammatory	
			Cooked with food, raw, infusion or eaten as a salad	Analgesic and anti-inflammatory	
			Poultice applied topically	Abscesses, acne	
			Poultice applied topically or infusion used orally	Rheumatism and arthralgia	
		Root		Rheumatism and arthralgia	
		Aerial part	Drink infusion with a daily break	Renal and urological disorders	
*Polygonum patulum* M.Bieb. (Polygonaceae)	Dermeği otu, kaşıntı otu/N	Aerial part	Juice used externally	Eczema and pruritic	0.05
*Portulaca oleracea* L. (Portulacaceae)	Pipirim/N	Aerial part	Boiled and eaten	Stomachaches and inflammations	0.03
				Asthma and dyspnea	
			Steam inhalation	Asthma and dyspnea	
			Boiled and eaten	Anticancer/prophylactic	
			Eaten as a salad		
			Infusion used orally		
*Prunus armeniaca* L. (Rosaceae)	Kayısı	Fruit	Eaten raw	Laxative	0.03
		Seed		Stomachaches and inflammations	
				Anthelmintic	
				Antidiarrheal	
*Prunus domestica* L. (Rosaceae)	Erik	Fruit	Mixture-5	Antidiarrheal	0.09
			Drinking as a Compote	Urological	
				Prostate	
*Prunus dulcis* (Mill.) D.A.Webb (Rosaceae)	Acıbadem, pisik payamı	Seed	Eaten raw	Analgesic	0.03
				Anthelmintic	
		Fruit, Flower		Antidiarrheal	
*Prunus hippophaeoides* (Bornm.) Bornm. (Rosaceae)	Kırmızı Dağ kirazı/N	Fruit	Eaten raw	Immunostimulant	0.09
*Prunus microcarpa* C.A.Mey. (Rosaceae)	Sarı dağ kirazı/N	Fruit	Eaten raw	Immunostimulant	0.09
*Prunus persica* (L.) Batsch (Rosaceae)	Şeftali	Fruit	Eaten raw	Antidiarrheal	0.02
		Flower	Infusion used orally	Antidiarrheal	
*Pulicaria dysenterica* Gaertn. subsp. *dysenterica* (Asteraceae)	-/N	Aerial part	Infusion used orally	Systemic inflammation	0.02
			Eaten raw	Systemic inflammation	
			Poultice applied topically	Topical anti-inflammatory	
*Quercus infectoria* Oliv. subsp. *infectoria* (Fagaceae)	Mazı meşesi/N	Seed	Dry powder applied to tooth	Toothache	0.05
*Rosa canina* L. (Rosaceae)	Gül burnu, Gül burcu, kuşburnu/N	Fruit	Decoction used orally	Common cold	0.68
				Analgesic	
				Immunostimulant	
			Jam used orally	Common cold	
				Analgesic	
				Immunostimulant	
*Salvia euphratica* Montbret & Aucher ex Benth. var. *euphratica* (Lamiaceae)	-/N	Leaf	Paste used externally	Eczema and pruritic	0.02
			Infusion used external	Eczema and pruritic	
*Salvia multicaulis* Vahl (Lamiaceae)	-/N	Leaf	Paste used externally	Eczema and pruritic	0.02
			Infusion used external	Eczema and pruritic	
*Salvia palaestina* Benth. (Lamiaceae)	Sığırdili/N	Leaf	Infusion used orally	Cardiovascular health	0.03
				Immunostimulant	
*Sambucus nigra* L. (Adoxaceae)	Paklanguç, Paklangıç/N	Flower	Infusion used orally	Asthma and dyspnea	0.19
				Common cold	
				Antitussive	
			Jam used orally	Asthma and dyspnea	
				Common cold	
				Antitussive	
			Dry powder applied externally	Wounds and burns	
*Satureja hortensis* L. (Lamiaceae)	Kekik/N	Aerial part	Eaten raw or dry	Nausea	0.06
				Stomachaches and inflammations	
*Solanum tuberosum* L. (Solanaceae)	Patates	Tuber	Topical application of slices	Headache and ocular pain	0.10
			Boiled and eaten	Antidiarrheal	
				Stomachaches and inflammations	
*Syzygium aromaticum* (L.) Merr. & L.M.Perry (Myrtaceae)	Karanfil	Bud	By sprinkling powder	Toothache	0.05
*Teucrium polium* L. subsp. *polium* (Lamiaceae)	-/N	Aerial part	Infusion used orally	Cardiovascular health	0.03
*Thymus kotschyanus* Boiss. & Hohen. (Lamiaceae)	Kekik/N	Aerial part	Eaten raw or dry	Stomachaches and inflammations	0.06
				Nausea	
*Thymus migricus* Klokov & Des.-Shost. (Lamiaceae)	Kekik/N	Aerial part	Eaten raw or dry	Nausea	0.06
				Stomachaches and inflammations	
*Tragopogon buphthalmoides* (DC.) Boiss. (Asteraceae)	Köse sakalı/N	Aerial part, Leaf	Eaten raw	Digestive	0.06
*Tragopogon dubius* Scop. subsp. *dubius* (Asteraceae)	Yemlik, Kargacık yemliği/N	Aerial part, Leaf	Eaten raw	Digestive	0.07
				Diabetes	
			Fungus-affected plants eaten raw	Anticancer/prophylactic	
*Tribulus terrestris* L. var. terrestris (Zygophyllaceae)	Çoban çökerten, çoban yastığı/N	Root	Decoction used orally	Asthma and dyspnea	0.02
*Tripleurospermum oreades* (Boiss.) Rech.f. (Asteraceae)	Dağ papatyası/N	Capitulum	Dry powder applied externally	Antiseptic	0.19
			Dry powder administered nasally	Epistaxis	
				Rhinorrhea	
				Ocular diseases	
			Infusion used orally	Common cold	
				Stomachache	
			Drink boiled with milk	Common cold	
				Stomachache	
*Triticum aestivum* L. (Poaceae)	Buğday	Seed	Dough applied topically	Traumatic injuries	0.18
				Topical anti-inflammatory	
			Mixture-8	Traumatic injuries	
		Straw	Mixture-10	Gynecological	
*Urtica dioica* L. subsp. *dioica* (Urticaceae)	Isırgan/N	Leaf	Externally	Rheumatism and arthralgia	0.34
			Infusion used orally	Urological	
				Prostate	
			Sitz bath with infusion	Hemorrhoids	
				Gynecological	
		Seed	Used orally with honey	Antitussive	
				Anticancer/prophylactic	
*Urtica urens* L. (Urticaceae)	Isırgan/N	Leaf	Externally	Rheumatism and arthralgia	0.34
			Infusion used orally	Urological	
				Prostate	
			Sitz bath with infusion	Hemorrhoids	
				Gynecological	
		Seed	Used orally with honey	Antitussive	
				Anticancer/prophylactic	
*Verbascum georgicum* Benth. (Scrophulariaceae)	Sığır kuyruğu sığır süpürgesi/N	Whole plant	Mixture-10	Gynecological	0.07
			Infusion used orally	For veterinary use	
		Flower	Decoction into a poultice and applied anally	Hemorrhoids	
		Leaf	Infusion used orally	Stomachaches and inflammations	
*Verbascum sphenandroides* K. Koch (Scrophulariaceae)	Sığır kuyruğu, sığır süpürgesi/N	Whole plant	Mixture-10	Gynecological	0.07
			Infusion used orally	For veterinary use	
		Flower	Decoction into a poultice and applied anally	Hemorrhoids	
		Leaf	Infusion used orally	Stomachaches and inflammations	
*Vicia hybrida* L. (Fabaceae)	Bacıt/N	Seed	Eaten raw	Immunostimulant	0.05
*Viola odorata* L. (Violaceae)	Menekşe/N	Flower	Infusion used orally	Common cold	0.03
*Vitis vinifera* L. (Vitaceae)	Üzüm, Asma	Fruit	Eaten raw	Immunostimulant	0.25
			Molasses, fruit leather, etc., used orally	Common cold	
				Infections	
			Molasses, fruit leather, etc., used topically	Infections	
			Mixture-8	Traumatic injuries	
			Mixture-11	Asthma and dyspnea	
				Jaundice	
		Stipes	Sap extract used orally	Smoking and alcohol cessation	

*: Use Values (UV), **: Native (N).

## Data Availability

The original contributions presented in this study are included in the article. Further inquiries can be directed to the corresponding author.

## Abbreviations

The following abbreviations are used in this manuscript:

BCE	Before Common Era
F_IC_	Informant Consensus Factor
GPS	Global Positioning System
ICF	Informant Consensus Factor
N	Number of informants
N_t_	Number of taxa
N_ur_	Number of use-reports
spp.	Species
UV	Use value

## Appendix A

### Literature Review

Ethnobotanical studies conducted in Türkiye prior have been reviewed. A table has been created for plants common to the Konak region, and information about their uses has been listed in the Appendix A (Table A1).

**Table A1:** Literature review.

Plant Name (Family)—Voucher No.	Literature Review	Used Part	References
*Ajuga chamaepitys* subsp. *chia* (Lamiaceae)- TK 1008	Anti-allergic, Antipyretic, Cough, Cystitis, Diabetes, Diuretic, Emenagog, Gynecological diseases, Hemorrhoid, Internal diseases, Stomachache, Strengthening agent, Urinary diseases	Aerial part	[19,42,43,44]
	Cough, Diabetes, Hemorrhoid, Healing wound, Wound healing	Flower	[21,44,45]
	Anti-allergic, Antipyretic, Cough, Cystitis, Diabetes, Diuretic, Emenagog, Gynecological diseases, Hemorrhoid, Strengthening agent, Urinary diseases	Leaves	[44]
*A. chamaepitys* subsp. *laevigata* (Lamiaceae)- TK 1007	Cough, Hemorrhoid, Stomachache, Wound healing	Aerial part	[43,44,46]
*Alcea apterocarpa* (Malvaceae)- TK 1223	Bronchitis, Cancer, Cough, Dyspnea, Gastrointestinal diseases, Inflammation, Infertility, Wound healing	Aerial part	[14,44]
	Bronchitis, Cough, Dyspnea,	Flower	[44,47],
	Bronchitis, Cough, Dyspnea,	Leaves	[44]
	Gastrointestinal diseases, Hemorrhoid, Inflammation, Wound healing	Root	[44]
*Allium ampeloprasum* (Amaryllidaceae)- TK 1115	Abscess, Blurred vision, Diabetes, Skin diseases, Strengthening, Wound healing	Bulb	[44,48]
	Abscess, Skin diseases, Wound healing,	Root	[44]
	Blurred vision, Diabetes,	Scape	[48]
	Abscess, Anticancer, Skin diseases, Wound healing,	Whole plant	[44,46]
*A. cepa* (Amaryllidaceae)- TK 1228	Abscess, Crushed, Inflammation, Scabies, Wound healing,	Bud	[44]
	Abdominal pain, Abortive, Abscess, Accelerate the menses, Analgesic, Antipyretic, Anuria, Arteriosclerosis, Bee bites, Bee sting, Bronchitis, Bruise, Carminative, Cicatrizing, Cough, Crushed, Diabetes, Digestive, Diuretic, Dysmenorrhea, Edema, Emanagog, Expectorant, Flu, Foot sprain, Fractured bones, Fracture, Gastric pain and ulcer, Gastric ulcers, Headache, Hemorrhoid, Hypercholesterolemia, Hypertension, Inflamed wound, Inflamed wounds, Inflammation, Kidney stone, Laxative, Maturation of abscess, Menstrual pain, Menstrual regulator, Muscular or rheumatic pain, Otitis, Paralysis, Prostatic diseases, Rheumatism, Scabies, Scorpion bites, Sprain, Stomachache, Swelling, Treat paralysis, Uterine inflammation, Uterus inflammation, Urinary inflammation, Whitlow, Wound healing, Wounds	Bulb	[14,15,18,42,43,44,46,49,50,51,52,53,54,55,56,57,58]
	Abortifacient, Dysuria,	İnner skin	[50]
	Abdominal pain, Arteriosclerosis, Carminative, Diabetes, Digestive, Gastric pain, Hemorrhoid, Hypercholesterolemia, Hypertension, Laxative, Ulcer,	Whole plant	[44]
	Abortive, Cough, Diuretic, Emanagog, Expectorant, Flu, Kidney stone, Menstrual pain, Menstrual regulator, Prostatic diseases, Uterine inflammation, Urinary inflammation	Leaves	[15,44]
	Appetizing, Diuretic,	Seed	[22]
*A. sativum* (Amaryllidaceae)- TK 1214	Abscess, Acne, Alopecia, Alopecia areata, Anthelmintic, Antihypertensive, Antiseptic, Appetizer, Arteriosclerosis, Bee bite, Cancer, Cardiac diseases, Carminative (for animal), Cardiovascular diseases, Cholesterol lowering, Cold, Diabetes, Diarrhea, Earache, Eczema, Erection, Erectile dysfunction, Expel worms, Flu, Food intoxication, Gastrointestinal diseases, Hair loss, Halitosis, Headache, Hemorrhoids, Hordeolum, Hypertension, Hypercholesterolemia, Hypotensive, Insect-bite, Lumbago, Malaria, Ovary diseases, Poisoning, Poisoning cattle, Rheumatic pain, Rheumatism, Ringworm, Scorpion bites, Scorpion poison, Skin Inflammation, Snake bite, Stye, Sunstroke, Tinea barbae, Tinea pedis, Whitlow, Wound healing, Worms	Bulbus	[18,37,42,44,46,48,49,50,51,52,53,54,55,57,58,59,60,61]
	Cold, Diabetes, Flu,	Flower	[48]
	Arteriosclerosis, Cancer, Cardiac diseases, Cardiovascular diseases, Cold, Diabetes, Diarrhea, Diuretic, Epilepsy, Flu, Gastrointestinal diseases, Hemorrhoid, Hypertension, Hypercholesterolemia, Kidney stone, Poisoning, Worms	Leaves	[44,48]
	Antihypertensive, Antiseptic, Appetizing, Heart regulation	Seed	[22]
	Arteriosclerosis, Cancer, Cardiac diseases, Cardiovascular diseases, Diarrhea, Diabetes, Gastrointestinal diseases, Hemorrhoid, Hypercholesterolemia, Hypertension, Poisoning, Worms	Whole plant	[44],
*Anchusa azurea* var. *azurea* (Boraginaceae)- TK 1088	Acne, Allergy, Antihypertensive, Burn, Carminative, Diabetes, Diaphoretic, Digestive, Diuretic, Eczema, Gastric pain, Gastric ulcer, Headache, Kidney diseases, Lip care, Rheumatism, Wound healing	Aerial part	[44,53,62]
	Burn, Diaphoretic, Eczema, Lip care, Stomachache, Stress, Tranquilizer, Wound healing	Flower	[20,26,36,44]
	Burn, Cold, Desiccant, Diaphoretic, Diuretic, Eczema, Flu, Gastric pain, Gastric ulcer, Hemostatic, Kidney diseases, Lip care, Rheumatism, Stomachache, Wound healing	Leaves	[15,18,19,20,26,42,44,46,54,63]
	Antihypertensive, Burn, Carminative, Diabetes disease, Diaphoretic, Digestive, Eczema, Lip care, Rheumatism, Wound healing	Root	[43,44,53]
*Anethum graveolens* (Apiaceae)- TK 1014	Aperitif, Antihypertensive, Carminative, Cholesterol, Digestive, Enteritis, Female diseases, Gastric diseases, Hiccough, Goiter, Hemorrhoids, Hypercholesterolemia, Kidney inflammation, Lactation, Strengthening	Aerial part	[44,64]
	Diuretic, Headache, Hemorrhoids, Hiccup, Appetizing, Galaktagog, Tranquilizer, Digestive, Carminative, Gastric diseases, Aperitif, Digestive, Enteritis, Strengthening, Halitosis, Hair loss, Female diseases, Lactation, Goiter, Hiccough	Leaves	[44,48,65]
	Cholesterol	Root	[64]
	Diuretic, Headache, Hemorrhoids, Hiccup	Seed	[48]
	Cholesterol	Whole plants	[64]
*Anthemis candidissima* (Asteraceae)- TK 1125	Diarrhea	Capitulum	[44],
*A. haussknechtii* (Asteraceae)- TK 1015	None	None	None
*Anthyllis vulneraria* subsp. *boissieri* (Fabaceae)- TK 1198	None	None	None
*Arnebia decumbens* (Boraginaceae)- TK 1004	None	None	None
*Astragalus gummifer* (Fabaceae)- TK 1035	Hemostatic,	Aerial part	[44]
	Throat diseases, Throat inflammation,	Latex	[20,26,44]
	Diabetes disease, Hemostatic, Throat diseases,	Root	[44,63]
*Bellis perennis* (Asteraceae)- TK 1123	Abscess, Acne, Bronchitis, Cold, Cough, Flu, Hair loss, Respiratory diseases, Shortness of breath, Throat pain, Tonsillitis	Aerial part	[44]
	Abscess, Acne, Abdominal pain, Antispasmodic, Antitussive, Bronchitis, Carminative, Carminative (baby), Cold, Colds, Cough, Diarrhea, Diuretic, Dyspnea, Eye diseases, Flu, Gastric pain, Gastric ulcer, Headache, Hemorrhoid, Hair loss, Kidney stone, Respiratory diseases, Shortness of breath, Stomachache, Stomachache (for children), Throat pain, Tonsillitis, Tranquilizer, Urinary diseases, Urinary inflammations	Capitulum	[15,18,20,21,26,44,54,65,66,67,68,69]
	Headache	Whole plants	[51]
*Brassica oleracea* (Brassicaceae)- TK 1213	Abdominal pain, Abscess, Cardiac diseases, Cough, Digestive, Earache, Enteric diseases, Fever, Gastric pain, Gastric ulcer, Headache, Hoarseness, Inflammatory eyelid, Kidney stone, Pain in stomach, Pneumonia, Rheumatism, Rheumatism inflammation, Ulcer, Urinary inflammation, Wound healing	Leaves	[26,43,44,50,51,55,56,57]
*Capsicum annuum* (Solanaceae)- TK 1235	Aperitif, Gastric pain, Gastric ulcer, Gastritis, Laxative, Stomachache	Fruit	[44,50]
*Cicer arietinum* (Fabaceae)- TK 1232	Kidney stones, Nephralgia, Ringworm treatment, Wart treatment	Fruit	[48]
	Diarrhea, Expel worms, Wound healing, Worms	Seed	[44,50]
*Citrus limon* (Rutaceae)- TK 1242	Acne, Antiemetic, Antihypertensive, Diuretic, Dandruff, Flu, Gastric pain, Headache, High fever, Kidney stone, Pneumonia,	Fruit	[44,50,51,60]
	Headache,	Leaves	[44,70]
	Headache,	Pericarp	[70]
*Cornus mas* (Cornaceae)- TK 1217	Antifungal, Antipyretic, Aphtha, Diarrhea, Itching, Rush, Tine pedis, Toothache, Wart	Barks	[44,52,65,71]
	Antihypertensive, Antipyretic, Aphrodisiac, Asthma, Bronchitis, Cardiac diseases, Cold, Cough, Diabetes, Diarrhea, Enteric pain, Flu, Gastric pain, Intestinal pain, Itching, Nephritis, Rush, Shortness of breath, Strengthening, Sun stroke, Sunstroke, Tinea pedis, Urinary inflammation, Wart	Fruits	[18,21,36,44,52,53,54,55,56,57,65,67,69,72,73]
	Abdominal pain, Antihypertensive, Bronchitis, Cardiac diseases, Cold, Cough, Diabetes, Diarrhea, Enteric pain, Flu, Gastric pain, Hypoglycemia, Shortness of breath, Urinary inflammation	Leaves	[44,53,55]
	Abdominal pain, Diabetes, Diarrhea, Enteric pain, Gastric pain, Intestinal pain, Sunstroke	Seed	[44,57,69]
*Crataegus monogyna* (Rosaceae)- TK 1093	Abdominal pain, Analgesic, Antihypertensive, Asthma, Bronchitis, Cardiac diseases, Cardiac disorder, Carminative, Cough, Diarrhea, Digestive, Gastric diseases, Gastric pain, Gastric ulcer, Headache, Heart diseases, Heart regulation, Hemorrhoid, Hypertensive, Laxative, Sedative, Stomachache, Stomach ailments, Tranquilizer, Urinary diseases, Vasodilator, Worm	Flowers	[15,18,22,23,26,44,52,69,72,74]
	Abdominal pain, Anti-spasmodic, Antihypertensive, Arteriosclerosis, Arythmia, Cardiac diseases, Cardiac disorder, Cardiovascular diseases, Cardiotonic, Carminative, Diabetes, Diarrhea, Digestive, Gastric diseases, Gastric pain, Gastric ulcer, Headache, Hemorrhoid, Hypotensive, Hypertensive, Kidney stone, Kidney stones, Laxative, Palpitation, Sedative, Spondylosis, Strengthening, Stomach ailments, Urinary diseases, Vasodilator, Worm	Fruits	[18,23,26,36,44,46,48,60,68,69,72]
	Abdominal pain, Analgesic, Antihypertensive, Arteriosclerosis, Arrythmia, Cardiac diseases, Cardiotonic, Carminative, Diabetes, Diarrhea, Digestive, Gastric diseases, Gastric pain, Gastric ulcer, Headache, Heart regulation, Hypertensive, Hypotensive, Laxative, Palpitation, Sedative, Tranquilizer, Vasodilator, Worm	Leaves	[22,23,44,46,48,69]
	Abdominal pain, Diarrhea, Digestive, Gastric diseases, Gastric pain, Gastric ulcer, Headache, Hemorrhoid, Laxative, Stomach ailments, Toothache, Urinary diseases, Worm,	Stipes	[44,72]
	Snake bite,	Thorns	[26,44,71]
	Bullet, Circulation problems, Heart disease, Nail, Sedative, Spasm, Heel spur, Wound	Root	[45]
*C. orientalis* subsp. *orientalis* (Rosaceae)- TK 1076	Rheumatism,	Cortex	[44]
	Antihypertensive, Bronchitis, Cardiac disorder, Heart diseases, Hemorrhoids, Kidney stone, Panacea, Stomachache, Stomach disorders, Vasodilator	Flower	[20,44,50,53,59,74]
	Cardiac disorder, Cardiotonic, Diabetes, Hemorrhoids, Kidney stone, Nephralgia, Panacea, Stomachache, Stomach disorders, Vasodilator	Fruit	[20,44,48,50,53]
	Cardiotonic, Diabetes, Kidney stones, Nephralgia, Vasodilator	Leaves	[48]
	Antihypertensive, Rheumatism,	Root	[14,44]
*Crocus damascenus* (Iridaceae)- TK 1158	None	None	None
*Cucumis sativus* (Cucurbitaceae)- TK 1231	Hypercholesterolemia, Jaundice, Kidney stone, Laxative, Nephrolithiasis prevention, Sunburn, Diuretic	Fruits	[44,51]
*Cyclotrichium niveum* (Lamiaceae)- TK 1054	Asthma, Tinea pedis,	Aerial part	[15,44]
	Asthma, Tinea pedis,	Leaves	[15,44]
*Cydonia oblonga* (Rosaceae)- TK 1219	Abdominal pain, Aperitif, Bronchitis, Cold, Cough, Diarrhea, Digestive, Enteric diseases, Expectorant, Flu, Gastric diseases, Gastric pain, Respiratory diseases, Shortness of breath, Throat diseases, Tonsillitis, Tuberculosis	Cortex	[44]
	Antihypertensive, Breast edema, Breast swelling, Cardiac diseases, Cardiotonic, Diabetes, Hemorrhoid, Hypercholesterolemia, Immunostimulant, Labor pain, Lactation, Stopping abortion, Uterine cancer,	Flower	[44]
	Abdominal pain, Aperitif, Asthma, Bronchial calmative, Bronchitis, Breast edema, Breast swelling, Cardiac diseases, Cardiotonic, Cold, Cough, Cystitis, Diabetes, Diarrhea, Digestive, Diuretic, Enteric diseases, Expectorant, Flu, Gastrointestinal diseases, Gastric diseases, Gastric pain, Hemorrhoid, Hypercholesterolemia, Immunostimulant, Lactation, Labor pain, Respiratory diseases, Shortness of breath, Stopping abortion, Throat diseases, Tonsillitis, Tuberculosis, Uterine cancer,	Fruits	[37,44,48,50,52]
	Abdominal ache, Abdominal pain, Aperitif, Antihypertensive, Antipyretic, Antitussive, Appetizer, Asthma, Breast edema, Breast swelling, Bronchitis, Cardiac diseases, Cardiotonic, Colds, Cold, Cough, Diabetes, Diabetes disease, Diarrhea, Diarrhea-animal, Diuretic, Digestive, Dysuria, Ease cough, Enteric diseases, Expectorant, Flu, Gastric diseases, Gastric pain, Gastrointestinal diseases, Headache, Hemorrhoid, Hemorrhoids, Hypercholesterolemia, Hypoglycemic, Immunostimulant, Influenza, Labor pain, Lactation, Respiratory diseases, Respiratory tract problem, Shortness of breath, Sore throat, Stomachache, Stomachache, Stopping abortion, Throat diseases, Tonsillitis, Tuberculosis, Uterine cancer, Urinary disorders, Wound healing,	Leaves	[15,37,43,44,48,50,51,52,53,55,56,57,59,60,61,67,69,71,72,73,74]
	Abdominal ache, Abdominal pain, Aperitif, Antihypertensive, Antipyretic, Antitussive, Appetizer, Asthma, Breast edema, Breast swelling, Bronchitis, Cardiac diseases, Cardiotonic, Colds, Cold, Cough, Diabetes, Diabetes disease, Diarrhea, Diarrhea-animal, Diuretic, Digestive, Dysuria, Ease cough, Enteric diseases, Expectorant, Flu, Gastric diseases, Gastric pain, Gastrointestinal diseases, Headache, Hemorrhoid, Hemorrhoids, Hypercholesterolemia, Hypoglycemic, Immunostimulant, Influenza, Labor pain, Lactation, Respiratory diseases, Respiratory tract problem, Shortness of breath, Sore throat, Stomachache, Stomachache, Stopping abortion, Throat diseases, Tonsillitis, Tuberculosis, Uterine cancer, Urinary disorders, Wound healing,	Seed	[44,52,69]
*Echinops pungens* (Asteraceae)- TK 1206	None	None	None
*Elaeagnus angustifolia* (Elaeagnaceae)- TK 1016	Abdominal pain, Choleric diseases, Diuretic, Dysurea, Enteric diseases, Gall bladder ailments, Gastric diseases, Gastric pain, Gastric ulcer, Hepatic diseases, Incontinence, Kidney diseases, Kidney stone, Kidney Stones, Laxative, Urinary diseases, Wart,	Cortex	[44,52,57]
	Abdominal pain, Amulet, Antipyretic, Aphrodisiac, As a vitamin, Blood purifying, Bronchitis, Choleric diseases, Cold, Diuretic, Enteric diseases, Fever, Flu, Gastric diseases, Gastric pain, Gastric ulcer, Hepatic diseases, Incontinence, Infertility, Kidney diseases, Kidney stone, Laxative, Nocturnal enuresis, Prostate disorders, Shortness of breath, Tonsillitis, Urinary diseases, Urinary incontinence	Flower	[15,24,44,45,46,65]
	Aphrodisiac, Abdominal pain, Antipyretic, As a vitamin, Asthma, Bronchitis, Carminative, Choleric diseases, Cold, Diabetes, Diuretic, Enteric diseases, Fever, Gall bladder ailment, Gastric diseases, Gastric pain, Gastric ulcer, Genital infections, Hepatic diseases, High cholesterol, Hypercholesterolemia, Incontinence, Infertility, Kidney ailment, Kidney diseases, Kidney stone, Kidney Stones, Laxative, Liver diseases, Nocturnal enuresis, pass kidney stone, Prostate disorders, Shortness of breath, Strengthening, Strengthening-Child, Sun stroke, Tonsillitis, Ulcer, Urinary tract infection, Urinary diseases, Urinary incontinence, Vision weakness, Wart	Fruit	[18,22,43,44,45,46,48,49,50,54,66,67,69]
	Abdominal pain, Antipyretic, Aphrodisiac, As a vitamin, Bronchitis, Choleric diseases, Cold, Diarrhea, Diuretic, Enteric diseases, Eye diseases, Fever, Gastric diseases, Gastric pain, Gastric ulcer, Hepatic diseases, Incontinence, Kidney diseases, Kidney stone, Laxative, Shortness of breath, To promote maturation of abscess, Tonsillitis, Urinary diseases,	Leaves	[36,42,44,45,59,60,69]
	Amulet, Blood purifying,	Root	[24]
	Abdominal pain, Choleric diseases, Enteric diseases, Gastric diseases, Gastric pain, Gastric ulcer, Hepatic diseases, Laxative	Seed	[44]
*Eminium intortum* (Araceae)- TK 1071	None	None	None
*Euphorbia* grisophylla (Euphorbiaceae)- TK 1005	Constipation,	Latex	[63]
*E. macrocarpa* (Euphorbiaceae)- TK 1138	Wound healing,	Latex	[44,64]
*Ficus carica* subsp. *rupestris* (Moraceae)- TK 1222	Abscess, Abdominal pain, Cancer, Callus treatment, Constipation, Diarrhea, Eczema, Flu, Gastric ulcer, Hemorrhoids, Hypercholesterolemia, Infertility, Inflamed breast, Laxative, Respiratory regulation, Skin inflammation, Skin tag, Stomach ulcer, Wart, Wound healing, Wounds	Fruits	[18,22,42,44,48,54,55,56,64,70,72,75]
	Abscess, Abdominal pain, Acne, Bee bites, Earache, Eczema, Hemorrhoids, Rhinorrhea, Scorpion bite, Skin inflammation, Skin tag, Stomach ulcer, Toothache, Wart, Wounds,	Latex	[14,42,43,44,49,57,59,61,66,71,72,73,75]
	Abscess, Abdominal pain, Asthma, Callus treatment, Cancer, Constipation, Eczema, Flu, Hemorrhoids, Infertility, Laxative, Skin inflammation, Skin tag, Stomachache, Wart,	Leaves	[44,48,54,55,60,61,71]
*Galium spurium* subsp. *ibicinum* (Rubiaceae)- TK 1140	None	None	None
*Geranium tuberosum* (Geraniaceae)- TK 1079	None	None	None
*Glycyrrhiza glabra* (Fabaceae)- TK 1237	Cancer, Wound healing,	Aerial part	[44]
	Analgesic, Bronchitis, Flu, Gastrointestinal diseases, Hemorrhoids, Infertility, Respiratory tract diseases, Smoking addiction, Sunstroke	Leaves	[43,44,48]
	Abdominal pain, Analgesic, Antihypertensive, Asthma, Bronchitis, Cardiac disorders, Cold, Cough, Diabetes mellitus, Diarrhea, Digestive disorders, Diuretic, Ease of cough, Enteric diseases, Epilepsy, Expectorant, Flu, Gastric diseases, Gastric pain, Gastric ulcer, Gastrointestinal diseases, Hemorrhoids, Hypercholesterolemia, Immunostimulant, Internal pain, Kidney diseases, Kidney stone, Oral disinfectant, Respiratory regulator, Respiratory tract diseases, Smoking addiction, Stomachache, Stomachic, Tranquilizer, Worm infestation	Root	[15,18,22,23,24,43,44,48,50,56,57,64,68]
*Gundelia tournefortii* (Asteraceae)- TK 1026	Appetizing, Cardiac diseases, Diabetes, Eczema, Kidney ache, Kidney stone, Kidney stones, Strengthening	Aerial part	[44,45]
	Aperitif, Aphrodisiac, Appetizing, Diabetes disease, Diarrhea, Dehydration, Digestive, Earache, Gastric diseases, Gingival strengthener, Kidney stones, Polydipsia, Strengthening gum, Tranquilizer	Latex	[15,18,20,26,44,45,50,53]
	Aphrodisiac, Cardiac diseases, Dermal allergic, Diabetes, Diabetes disease, Diarrhea, Digestive, Impotence, Jaundice, Kidney ache, Kidney stone, Prolonged menstrual bleeding, Vaginal discharges	Root	[18,44,46,53]
	Cold, Vitiligo,	Seed	[44,68]
*Helichrysum arenarium* (Asteraceae)- TK 1038	Cancer, Diabetes disease, Diuretic, Gastrointestinal diseases, Gynecological diseases, Hepatitis, Hypercholesterolemia, Itching, Kidney stones, Nephralgia, Stomach ulcer, Wound healing	Aerial part	[21,24,44,48,63]
	Abdominal pain, Asthma, Carminative, Cough, Diarrhea, Diuretic, Earache, Gastric ulcer, Gynecologic diseases, Inflammation, Itching, Kidney diseases, Kidney gravel, Kidney stones, Nephralgia, Spontaneous kidney stone and sand passage, Stomach diseases, Stomachache, Ulcer, Wound healing	Capitulum	[23,44,45,47,65,66,74]
	Carminative, Diarrhea, Gynecologic diseases, Inflammation, Kidney diseases	Leaves	[23]
*H. plicatum* subsp. *plicatum* (Asteraceae)- TK 1043	Abdominal pain, Accelerate blood circulation, Cholagogue, Cold, Cough, Depreciatory, Diabetes, Diuretic, Earache, Earache (for baby), Flu, Gastric pain, Gastric ulcer, Hemostatic, Hypercholesterolemia, Jaundice, Kidney and stomach ailments, Kidney stone, Kidney stones, Larynx cancer, Prostatic diseases, Rheumatism, Sedative, Shortness of breath, Snake repellent, Stomachache, Stomach ulcer, Vascular diseases	Aerial part	[14,19,21,24,44,45,54,64]
	Abdominal pain, Accelerate blood circulation, Anti-fungal, Cholagogue, Cold, Cough, Diabetes, Diabetes disease, Diuretic, Gastric pain, Gastric ulcer, Hepatitis, Hypercholesterolemia, Jaundice, Kidney Stones, Larynx Cancer, Piles on hand and foot, Shortness of breath, Urinary dysfunction, Vascular diseases, Wound healing	Capitulum	[15,20,22,44,50,53,57,63,68]
*Hordeum vulgare* (Poaceae)- TK 1233	Anorexia, Appetizing, Diarrhea, Intestinal regulation	Aerial part	[48]
	Abscess, Bruising, Itching, Tinea pedis, Wart, Wound healing,	Flour	[44]
	Abdominal pain, Anorexia, Appetizing, Antipyretic, Bruising, Cough, Cough in pneumonia, Cold, Common cold, Diabetes, Diuretic, Dermatophytes, Edema (animal), Facial paralysis, Gonorrhea, Headache, Hypercholesterolemia, Immunostimulant, Intestinal regulation, Itching, Kidney inflammation, Kidney stone, Kidney stones, Nephritis, Rheumatic pain, Rheumatism, Skin inflammation, Strengthening, Tinea pedis, Tuberculosis, Urinary inflammation, Uterine inflammation, Wound healing	Seed	[18,20,26,44,48,50,52,55,56,57]
*Juglans regia* (Juglandaceae)- TK 1221	Abscess, Acne, Aperitif, Arteriosclerosis, Barber’s itch, Bleeding, Bronchitis, Cancer, Cardiac diseases, Cold, Cough, Dandruff, Diabetes, Diaphoretic, Digestive disorders, Eczema, Enteric laxative, Erysipeloid, Expectorant, Flu, Foot odor, Foot sweat, Freckle, Fungal diseases, Galactagogue, Gastric pain, Gonorrhea, Hair care, Hair loss, Hand care, Hemorrhoid, Hemostatic, Herpes, Hypercholesterolemia, Hypertensive, Hypoglycemic, Infertility, Malaria, Menstrual pain, Psoriasis, Promoter of abscess maturation, Respiratory diseases, Rheumatism, Scabies, Shortness of breath, Skin diseases, Skin inflammation, Strengthening agent, Syphilis, Throat diseases, Throat pain, Tinea pedis, Tonsillitis, Vaginal inflammation, Wart, Whopping cough, Wound antiseptic, Wound healing, Worm infestation	Cortex	[44,60,65,68]
	Abscess, Acne, Analgesic, Antidiaphoretic, Antifungal, Antipyretic, Antiseptic, Antidiabetic, Aperitif, Aphtha, Arteriosclerosis, Arthralgia, Barber’s itch, Bee bites, Bleeding, Bronchitis, Cancer, Cardiac diseases, Cardiac disorder, Cardio tonic, Cholesterol lowering agent, Cold, Consciousness-expanding, Cough, Cystitis, Dandruff, Diaphoretic, Diarrhea, Diabetes, Digestive disorders, Edema, Enteric laxative, Eczema, Expectorant, Eye pain, Erysipelas, Erysipeloid, Fever, Flu, Foot odor, Foot sweat, Fracture, Freckle, Fungal diseases, Fungal infection, Gastric pain, Gingival diseases, Goiter, Gonorrhea, Hair care, Hair loss, Hair restorer, Headache, Hemorrhoid, Hemostatic, Herpes, High cholesterol, Hypercholesterolemia, Hypertensive, Hypoglycemic, Injury, Infertility, Kidney diseases, Kidney stone, Malaria, Memory improvement, Menstrual pain, Mouth ulcer, Nosebleed, Osteoporosis, Pain, Pimples, Promoter of maturation of abscess, Psoriasis, Respiratory diseases, Respiratory tract problem, Rheumatic pain, Rheumatism, Sore throat, Scabies, Skin diseases, Skin inflammation, Slimming agent, Sprain, Stomachache, Strengthening agent, Sunstroke, Syphilis, Tension regulator, Throat diseases, Throat pain, Tinea pedis, Tonic, Tonsillitis, Toothache, Tranquilizer, Urinary diseases, Vaginal inflammation, Vaginitis, Vasodilator, Wart, Whopping cough, Women’s sterility, Worm infestation, Wound, Wound antiseptic, Wound healing	Fruit	[14,15,18,19,26,42,44,45,46,48,50,51,52,53,54,55,57,61,63,64,66,67,68,69,71,72,73,74],
	Abscess, Acne, Analgesic, Antidiaphoretic, Antifungal, Antipyretic, Antiseptic, Antidiabetic, Aperitif, Aphtha, Arteriosclerosis, Arthralgia, Bee bites, Bleeding, Bronchitis, Cancer, Cardiac diseases, Cardio tonic, Cough, Cystitis, Dandruff, Diarrhea, Diaphoretic, Diabetes, Digestive, Edema, Enteric laxative, Epistaxis, Eczema, Expectorant, Eye pain, Female infertility, Fever, Flu, Foot odor, Foot sweat, Freckle, Fracture, Fungal diseases, Fungal infection, Gastric pain, Gingival diseases, Goiter, Gonorrhea, Hair care, Hair loss, Hair restorer, Headache, Hemorrhoid, Hemostatic, Herpes, Hypercholesterolemia, Hyper cholesterol, Hypertensive, Hypoglycemic, Injury, Infertility, Kidney diseases, Kidney stone, Malaria, Memory improvement, Menstrual pain, Mouth and nostril wounds, Pain, Pimples, Psoriasis, Promoter of maturation of abscess, Respiratory diseases, Rheumatic pain, Rheumatism, Scabies, Skin diseases, Skin inflammation, Slimming agent, Sprain, Stomachache, Strengthening agent, Sunstroke, Syphilis, Tension regulator, Throat diseases, Throat pain, Tinea pedis, Tonsillitis, Toothache, Galaktagog,, Urinary diseases, Vaginal inflammation, Vaginitis, Vasodilator, Wart, Whopping cough, Wound, Wound antiseptic, Wound healing, Worms,	Leaves	[15,22,23,36,42,43,44,46,47,48,50,52,55,56,57,58,59,61,65,67,68,69,70,71,72,74,75]
	Arteriosclerosis, Arthralgia, Cancer, Cardiac diseases, Diabetes, Edema, Fracture, Hemorrhoid, Hemostatic, Hypercholesterolemia, Hypertensive, Injury, Rheumatism, Slimming agent, Sprain	Roots	[43,44,52]
*Malus domestica* (Rosaceae)- TK 1229	Antipyretic, Dyspepsia,	Fruit	[44,57]
	Scorpion bite,	Leaves	[42]
*Malva neglecta* (Malvaceae)- TK 1059	Abdominal pain, Abortion, Abscess, Acne, Amenorrhea, Anemia, Anti-inflammatory, Anti-hypertension, Antipyretic, Antitussive, Aphonia, Aphtha, Arthralgia, Asthma, Boil, Bronchitis, Cancer, Cardiac diseases, Chest laxative, Cold, Constipation, Cough, Debilitating, Diabetes, Diuretic, Digestive, Eczema, Edema, Expectorant, Female diseases, Flu, Gastrointestinal diseases, Gastrointestinal inflammation, Gingivitis, Goiter, Headache, Hemophilia, Hemorrhoid, Hemorrhoids, Hepatic diseases, Hoarseness, Hypercholesterolemia, Immunostimulant, Infertility, Injury, Kidney ache, Kidney diseases, Kidney inflammation, Kidney stone, Kidney stones, Laxative, Liver diseases, Lumbago, Menstrual diseases, Menstrual pain, Mumps, Myalgia, Nausea, Pain, Parotitis, Prostatic diseases, Respiratory diseases, Rheumatism, Shortness of breath, Skin inflammation, Sore throat, Stomachache, Stomach diseases, Stomach ulcer, Throat diseases, Throat pain, Tonsillitis, Toothache, Tranquilizer, Ulcer, Urinary diseases, Urinary inflammation, Urticaria, Uterine diseases, Vaginal discharge, Wart, Wound healing, Wound pain, Wounds,	Aerial part	[14,15,18,20,21,26,42,44,50,53,54,56,62,63,64,76]
	Abscess, Acne, Antipyretic, Bronchitis, Bruising, Cancer, Cardiac diseases, Chest laxative, Cold, Cough, Diabetes, Diuretic, Eczema, Expectorant, Female diseases, Gastrointestinal diseases, Hoarseness, Hypercholesterolemia, Immunostimulant, Infertility, Menstrual pain, Psoriasis, Prostatic diseases, Respiratory diseases, Respiratory inflammation, Shortness of breath, Skin diseases, Skin inflammation, Stomachache, Stuffiness, Throat diseases, Throat pain, Tonsillitis, Tuberculosis, Urinary inflammation, Urinary diseases, Vaginal inflammation, Wound, Wound healing,	Flower	[44,48,72]
	Abdominal pain, Abortifacient, Abortive, Abscess, Acne, Aphtha, Anti-hypertension, Antipyretic, Arteriolosclerosis, Arthralgia, Asthma, Boil, Bronchitis, Bruise, Bruising, Cancer, Carminative, Cardiac diseases, Chest laxative, Choleretic, Cold, Constipation, Cough, Diabetes, Diuretic, Digestive disorders, Edema, Eczema, Emanagog, Enteritis, Expectorant, Female diseases, Fracture, Gastrointestinal diseases, Gastric diseases, Gastric pain, Gastric ulcer, Gingivitis, Headache, Hemorrhoid, Hemostatic, Hepatic diseases, Hoarseness, Hypercholesterolemia, Immunostimulant, Infertility, Injury, Intestinal diseases, Kidney ache, Kidney diseases, Kidney inflammation, Kidney stone, Laxative, Maturation of abscess, Menstrual pain, Oral pain, Promoter of abscess maturation, Prostatic diseases, Psoriasis, Respiratory diseases, Respiratory inflammation, Rheumatism, Shortness of breath, Skin diseases, Skin inflammation, Sterility, Stomachache, Stuffiness, Throat diseases, Throat pain, Tonsillitis, Tuberculosis, Urinary diseases, Urinary inflammation, Vaginal inflammation, Wound, Wound healing	Leaves	[19,24,43,44,45,47,48,50,54,57,59,63,64,66,68,72,74]
	Abortifacient, Abortive, Abscess, Acne, Antipyretic, Bronchitis, Bruising, Cancer, Chest laxative, Cold, Cough, Diuretic, Eczema, Emanagog, Expectorant, Female diseases, Hoarseness, Infertility, Kidney ache, Kidney diseases, Kidney inflammation, Kidney stone, Menstrual pain, Psoriasis, Prostatic diseases, Respiratory diseases, Respiratory inflammation, Shortness of breath, Skin diseases, Skin inflammation, Stuffiness, Throat diseases, Throat pain, Tonsillitis, Tuberculosis, Urinary diseases, Urinary inflammation, Vaginal inflammation, Wound, Wound healing,	Root	[44,45,50]
*M. sylvestris* (Malvaceae)- TK 1191	Abdominal pain, Abortion, Abscess, Acne, Amenorrhea, Anemia, Anti-inflammatory, Anti-hypertension, Antipyretic, Antitussive, Aphonia, Aphtha, Arthralgia, Asthma, Boil, Bronchitis, Cancer, Cardiac diseases, Chest laxative, Cold, Constipation, Cough, Debilitating, Diabetes, Diuretic, Digestive, Eczema, Edema, Expectorant, Female diseases, Flu, Gastrointestinal diseases, Gastrointestinal inflammation, Gingivitis, Goiter, Headache, Hemophilia, Hemorrhoid, Hemorrhoids, Hepatic diseases, Hoarseness, Hypercholesterolemia, Immunostimulant, Infertility, Injury, Kidney ache, Kidney diseases, Kidney inflammation, Kidney stone, Kidney stones, Laxative, Liver diseases, Lumbago, Menstrual diseases, Menstrual pain, Mumps, Myalgia, Nausea, Pain, Parotitis, Prostatic diseases, Respiratory diseases, Rheumatism, Shortness of breath, Skin inflammation, Sore throat, Stomachache, Stomach diseases, Stomach ulcer, Throat diseases, Throat pain, Tonsillitis, Toothache, Tranquilizer, Ulcer, Urinary diseases, Urinary inflammation, Urticaria, Uterine diseases, Vaginal discharge, Wart, Wound healing, Wound pain, Wounds,	Aerial part	[23,37,44,48,52,53,62,67,69,70,71,72,74,76]
	Abdominal pain, Abortive, Acne, Antihypertensive, Antipyretic, Antitussive, Bronchitis, Cancer, Cardiac diseases, Chest laxative, Cold, Cough, Diarrhea, Digestive, Diabetes, Diuretic, Eczema, Edema, Expectorant, Female diseases, Flu, Furuncle, Galaktagog, Gastrointestinal diseases, Gastrointestinal inflammation, Gastric diseases, Gastric pain, Goiter, Headache, Hemophilia, Hemorrhoid, Hemorrhoids, Hepatic diseases, Hoarseness, Hypercholesterolemia, Immunostimulant, Infertility, Injury, Intestinal disease, Kidney ache, Kidney diseases, Kidney inflammation, Kidney sand, Kidney stone, Laxative, Liver diseases, Lumbago, Menstrual diseases, Menstrual pain, Mumps, Myalgia, Nausea, Pain, Parotitis, Prostatic diseases, Respiratory diseases, Rheumatism, Scorpion bite, Skin inflammation, Stomachache, Stomach diseases, Stomach ulcer, Throat diseases, Throat pain, Tonsillitis, Toothache, Tranquilizer, Urinary diseases, Urinary inflammation, Urticaria, Uterine diseases, Vaginal discharge, Wart, Wound, Wound healing, Wound pain	Flowers	[44,49,52,61,67,70,71,72,73]
	Abdominal pain, Abortive, Acne, Antihypertensive, Antipyretic, Anti-inflammatory, Boil, Bronchitis, Cancer, Cardiac diseases, Cold, Constipation, Cough, Debilitating, Diabetes, Diuretic, Digestive, Eczema, Edema, Expectorant, Female diseases, Flu, Gastrointestinal diseases, Gastrointestinal inflammation, Gastric diseases, Gastric pain, Goiter, Headache, Hemophilia, Hemorrhoid, Hepatic diseases, Hoarseness, Hypercholesterolemia, Inflammation, Infertility, Injury, Kidney ache, Kidney diseases, Kidney inflammation, Kidney stone, Laxative, Liver diseases, Menstrual diseases, Menstrual pain, Myalgia, Nausea, Pain, Parotitis, Prostatic diseases, Respiratory diseases, Rheumatism, Skin inflammation, Sore throat, Stomachache, Stomach ulcer, Throat diseases, Throat pain, Tonsillitis, Toothache, Uterine diseases, Vaginal discharge, Wound healing, Wound pain,	Leaves	[37,44,46,49,52,58,61,66,67,69,70,72,73]
	Abdominal pain, Abortive, Acne, Antihypertensive, Antipyretic, Cancer, Cardiac diseases, Cold, Constipation, Diabetes, Digestive, Diuretic, Eczema, Edema, Female diseases, Gastrointestinal diseases, Gastrointestinal inflammation, Gastric diseases, Gastric pain, Headache, Hemorrhoid, Hypercholesterolemia, Infertility, Kidney ache, Kidney diseases, Kidney inflammation, Kidney stone, Liver diseases, Menstrual diseases, Menstrual pain, Pain, Prostatic diseases, Rheumatism, Skin inflammation, Stomachache, Tonsillitis, Toothache, Uterine diseases, Vaginal discharge, Wound healing, Wound pain,	Roots	[44,48,49,52,62,72]
	Cold,	Seeds	[70]
*Mentha longifolia* subsp. *longifolia* (Lamiaceae)- TK 1003	Abdominal pain, Antiseptic, Bronchitis, Carminative, Cold, Colds, Cough, Diarrhea, Digestive disorders, Flu, Gastric diseases, Gastric pain, Headache, Hemorrhoid, High fever, Internal diseases, Lung diseases, Menstrual pain, Nausea, Shortness of breath, Stomach, Stomachache, Sunstroke, Tachycardia, Worm infestation	Aerial part	[22,36,42,44,50,56,57,59,63,64]
	Abdominal pain, Aphtha, Anti-allergic, Bronchitis, Cardiac arrhythmia, Cold, Cough, Diarrhea, Diuretic, Digestive disorders, Gastric diseases, Gastric pain, Hair loss, Heart palpitation, Hemorrhoid, Kidney stone, Lung diseases, Nausea, Rheumatism, Shortness of breath, Stomachache, Styptic, Tachycardia, Worm infestation,	Leaves	[44,45,52,55,62,63,68,74]
	Headache	Root	[44]
*M. pulegium* (Lamiaceae)- TK 1052	Abdominal pain, Anti-allergic, Anti-asthmatic, Aperitif, Appetizer, Bronchitis, Cardiac diseases, Carminative, Choleretic diseases, Cold, Colds, Cough, Diabetes, Diuretic, Digestive, Flu, Gall bladder disorders, Gastric diseases, Gastric pain, Heatstroke, Herpes, Intestinal diseases, Itching, Kidney diseases, Laxative, Malaria, Nausea, Poisoning, Respiratory antiseptic, Rheumatism, Shortness of breath, Snakebite envenomation, Stimulant, Stomachache, Stomach diseases, Stomachic, Sunburn, Throat pain, Tranquilizer	Aerial part	[36,44,57,59,67,68,70,76]
	Abdominal pain, Analgesic, Aperitif, Bronchitis, Carminative, Choleretic diseases, Cold, Cough, Digestive, Flu, Gall bladder disorders, Gastric diseases, Gastric pain, Headache, Malaria, Migraine, Nausea, Poisoning, Respiratory antiseptic, Shortness of breath, Throat pain, Intestinal diseases	Flower	[44,68]
	Abdominal pain, Analgesic, Anti-Allergic, Antiemetic, Aperitif, Bronchitis, Cardiac diseases, Carminative, Carminative (for babies), Choleretic diseases, Cold, Cough, Diabetes, Digestive, Diuretic, Emanagog, Flu, Gastric diseases, Gastric pain, Headache, Herpes, Influenza, Intestinal diseases, Itching, Kidney diseases, Menstruation, Migraine, Malaria, Nasal congestion, Nausea, Poisoning, Respiratory antiseptic, Rheumatism, Rhinocleisis, Shortness of breath, Stomachache, Stomachic, Sunburn, Throat pain	Leaves	[44,54,60,67,69]
*M. spicata* subsp. *spicata* (Lamiaceae)- TK 1039	Antispasmodic, Aperitif, Aphthae (baby), Arthralgia, Bronchitis, Carminative, Cold, Cough, Diarrhea, Digestive, Diuretic, Flu, Gastric diseases, Gastric pain, Halitosis, Headache, Intestinal pain, Lactation stimulant, Malaria, Myalgia, Nausea, Prostatic diseases, Respiratory diseases, Shortness of breath, Stomach-ache, Sunstroke, Throat diseases, Tonic, Urinary diseases, Vomit,	Aerial part	[19,20,26,36,44,57,71]
	Aperitif, Aphthae (for babies), Appetite, Antispasmodic, Bronchitis, Carminative, Cold, Colds and flu, Cough, Diabetes, Diarrhea, Digestive, Flu, Gastric diseases, Gastric pain, Hair loss, Halitosis, Hemorrhoid, Intestinal pain, Jaundice, Lactation stimulant, Malaria, Nausea, Prostatic diseases, Respiratory diseases, Respiratory problem, Rheumatism, Shortness of breath, Sickness, Stomachache, Strengthening, Throat diseases, Toothache, Tranquilizer, Vomit,	Leaves	[15,18,26,44,53,59,60,66,70]
*Morus alba* (Moraceae)- TK 1224	Abdominal pain, Abscess, Anemia, Aphtha, Aperitif, Cancer, Cardiac diseases, Chest laxative, Cold, Cough, Diabetes, Diuretic, Eczema, Gastric diseases, Gastric pain, Gastric ulcer, Hepatic diseases, Hypercholesterolemia, Hypoglycemia, Intestinal diseases, Laxative, Maturation of abscess, Oral candidiasis (babies), Reddening of the eyes, Shortness of breath, Stomach disorders, Strengthening, Thrush, Toothache, Urinary diseases, Urinary inflammation,	Fruits	[18,19,26,42,44,54,55,56,59,69,73,74,76]
	Abdominal pain, Abscess, Anemia, Anthelmintic, Antipyretic, Aphtha, Aperitif, Bronchitis, Cancer, Cardiac diseases, Chest laxative, Cold, Cough, Diabetes, Diuretic, Epistaxis, Eczema, Gastric diseases, Gastric pain, Gastric ulcer, Hepatic diseases, Hypercholesterolemia, Hypoglycemia, Intestinal diseases, Nosebleed, Panaris, Shortness of breath, Toothache, Urinary diseases, Urinary inflammation, Whitlow,	Leaves	[15,19,22,44,46,67,71,74]
*M. nigra* (Moraceae)- TK 1212	Amenorrhea, Anemia, Cancer, Cure baldness, Diabetes, Dysmenorrhea, Eczema, Emanagog, Hair loss, Hemorrhoid, Hemorrhoids, Herpes infection, Hypercholesterolemia, Kidney diseases, Kidney stone, Skin diseases, Stomachache, Trush, Urinary diseases, Wound healing	Bark	[44,55,57,69,76]
	Aphtha, Aphtha (baby), Aphtha (repeated), Anemia, Asthma, Blood forming, Cancer, Cholesterol, Cure baldness, Diabetes, Eczema, Fever, Gastric pain, Gout, Hair loss, Handicrafts, Headache, Hepatic diseases, Herpes infection, Hypercholesterolemia, Hypoglycemic, Jaundice, Mouth diseases, Respiratory regulation, Skin diseases, Stomachache, Strengthening, Trush, Wound healing, Worms	Fruits	[22,36,42,44,50,52,53,54,57,58,59,72,74,76]
	Anemia, Antipyretic, Aphtha, Cancer, Diabetes, Eczema, Gastric pain, Hair loss, Hepatic diseases, Hemorrhoid, Hypercholesterolemia, Jaundice, Kidney diseases, Kidney stone, Shortness of breath, Skin diseases, Throat diseases, Throat pain, Tonsillitis, Urinary diseases, Worms, Wound healing,	Leaves	[24,44,50,52,70,74]
	Abortifacient, Abortive, Diabetes, Emanagog, Fever, Gastric pain, Hepatic diseases, Jaundice, Respiratory regulation, Worms,	Root	[22,44,50]
*Olea europaea* (Oleaceae)- TK 1240	Arthralgia, Diabetes, Earache, Nausea, Stomachache, Toothache	Cortex	[36]
	Abscess, Analgesic, Antipyretic, Arthralgia, Bronchitis, Burn, Cancer, Cardiotonic, Carminative, Cold, Constipation, Cough, Cuts, Diabetes, Earache, Eczema, Eye diseases, Gastric pain, Gingival diseases, Hair loss, Hemorrhoid, Hypercholesterolemia, Hypertension, Kidney ache, Kidney stone, Myalgia, Nausea, Rash, Rheumatism, Scorpion bite, Shortness of breath, Skin care, Sore throat, Sprain, Stomachache, Swelling, Tachycardia, Throat diseases, Tonsillitis, Toothache, Vasodilator, Wen, Whooping cough, Wound, Wound healing	Fruit	[36,43,44,48,50,52,55,56,57,72]
	Abscess, Alopecia, Antihypertensive, Antipyretic, Appetizing, Aphtha, Arthralgia, Bronchitis, Bruising, Burn, Cancer, Cardiotonic, Cold, Constipation, Cuts, Depressor, Diabetes, Earache, Eczema, Eye diseases, Gingival diseases, Hair loss, Hemorrhoid, Hypercholesterolemia, Hypertension, Kidney ache, Kidney stone, Myalgia, Nausea, Nodules, Rash, Rheumatism, Shortness of breath, Skin care, Sore throat, Sprain, Stomachache, Swelling, Tachycardia, Throat diseases, Toothache, Tonsillitis, Vasodilator, Wen, Whooping cough, Wound, Wound healing,	Leaves	[22,23,36,44,48,51,56,60,61,70,72]
	Abscess, Antipyretic, Arthralgia, Asthma, Bee bites, Bronchitis, Bruising, Burn, Cancer (gastrointestinal), Cardiotonic, Carminative, Cold, Development of bones, Earache, Eczema, Eye diseases, Hair loss, Hemorrhoid, Hypercholesterolemia, Hypertension, Kidney ache, Kidney stone, Myalgia, Rash, Rheumatism, Snake bites, Shortness of breath, Sore throat, Sprain, Swelling, Throat diseases, Tonsillitis, Vasodilator, Whooping cough, Wound, Wound healing,	Oil	[44,51,59]
	Abscess, Analgesic, Antipyretic, Arthralgia, Bronchitis, Bruising, Cancer, Cardiotonic, Cold, Cuts, Diabetes, Earache, Eczema, Hair loss, Hemorrhoid, Hypercholesterolemia, Hypertension, Kidney ache, Kidney stone, Myalgia, Rash, Rheumatism, Scorpion bite, Shortness of breath, Sore throat, Sprain, Swelling, Tachycardia, Throat diseases, Tonsillitis, Toothache, Vasodilator, Wen, Whooping cough, Wound, Wound healing	Seed	[44,48,50,52]
*Origanum vulgare* subsp. *gracile* (Lamiaceae)- TK 1053	Cold, Diabetes, Diuretic, Epilepsy, Flu, Gastric diseases, Gastric pain, Gingivitis, Headache, Jaundice, Nausea, Stomachache, Stomach ailments, Stomach diseases, Strengthening the bones, Toothache, Urinary inflammation, Wound, Wound healing	Aerial part	[36,42,44,45,52,59,68,69,71,75]
	Antihypertensive, Cold, Colds and flu, Diabetes, Diuretic, Digestive, Epilepsy, Flu, Gastric diseases, Gastric pain, Headache, Hemorrhoids, Hepatitis, Hypercholesterolemia, Hypertension, Jaundice, Nausea, Sedative, Shortness of breath, Stomachache, Toothache, Tranquilizer, Urinary inflammation	Leaves	[18,26,36,44,53,54,60,75]
	Cold, Diabetes, Flu, Gastric diseases, Gastric pain, Headache, Hypertension, Jaundice, Nausea, Shortness of breath, Toothache		[44]
*Pelargonium endlicherianum* (Geraniaceae)- TK 1124	Anthelminthic,	Aerial part	[44],
	Anthelminthic,	Flower	[15,22,44]
	Anthelminthic,	Leaves	[15,22,44]
	Anthelminthic,	Root	[50],
*Petroselinum crispum* (Apiaceae)- TK 1216	Abdominal ache, Abdominal pain, Anemia, Anti aggregate, Anti hypertension, Common cold, Diabetes, Diuretic, Digestive, Eczema, Gastric diseases, Halitosis, Hemorrhoid, Hepatic diseases, Hypercholesterolemia, Kidney ache, Shortness of breath, Stomach disorders	Aerial part	[37,44,51,55]
	Abdominal pain, Acne, Anemia, Anti aggregate, Anti-hypertension, Antidiabetic, Aphrodisiac, Common cold, Cystitis, Diabetes, Diuretic, Digestive, Edema, Emanagog, Eczema, Eye diseases, Female inflammation, Galaktagog, Gastric diseases, Gastritis, Halitosis, Hemorrhoid, Hepatic diseases, Hypercholesterolemia, Injury, Kidney ache, Kidney diseases, Kidney stone, Kidney stones, Laxative, Lumbago, Mouth sores, Shortness of breath, Slimming down, Sprain, Stomach pains, Syphilis, Ulcer, Urinary diseases, Urinary inflammation, Vaginal discharge	Leaves	[18,22,44,46,54]
	Anemia, Anti aggregate, Anti hypertension, Diabetes, Diuretic, Hemorrhoid, Hypercholesterolemia, Kidney diseases, Kidney stone, Mouth sores, Urinary diseases,	Roots	[18,44,51,54]
	Heart diseases, Kidney stone,	Seed	[23]
*Pistacia terebinthus* (Anacardiaceae)- TK 1236	Antiseptic, Diarrhea, Expectorant, Foot sweating, Gastric diseases, Gastric pain, Gastric ulcer, Gastritis, Kidney Stones, Stomachache, Stomach pains, Ulcer	Branches	[15,44,46,52,53]
	Aphrodisiac, Appetizer, Antiseptic, Asthma, Cancer, Consciousness-expanding, Diarrhea, Diabetes, Diuretic, Expectorant, Foot sweating, Gastric diseases, Gastric pain, Gastric ulcer, Gastritis, Gingivitis, heart disease, heat stroke, Hemorrhoid, Kidney Stones, Stimulant, Stomach-ache, Stomachache, Stomach pains, Stomach ulcers, Ulcer, Urinary inflammations	Fruits	[18,20,26,43,44,46,48,50,53,60,72]
	Antiseptic, Asthma, Gingivitis, Stomachache, Wound healing	Gum	[22,48,60]
	Wound healing –animal	Latex	[44]
	Antifungal, Antiseptic, Cancer, Diabetes, Diarrhea, Gastric diseases, Gastric pain, Gastric ulcer, Hemorrhoids, Mycosis, Peptic ulcer, Psoriasis, Shortness of breath, Sterility, Stomachache, Stomachache, Sunstroke	Leaves	[15,42,44,49,58,67,68]
*Plantago lanceolata* (Plantaginaceae)- TK 1001	Arteriolosclerosis, Bronchitis, Cardiovascular diseases, Cough, Diabetes, Diarrhea (children), Digestive, Embolism, Expectoration, Gastric diseases, Gastric pain, Gastric ulcer, Gastritis, Hemorrhoid, Hemostatic, Hepatic diseases, Laxative	Aerial part	[44,52,60,71]
	Abscess, Acne, Antipyretic, Astringent, Bee bites, Blood purification, Boil, Burn, Cardiovascular diseases, Cough, Cystitis, Cuts, Diabetes, Diuretic, Digestive, Eczema, Embolism, Expectorant, Furuncle, Gastric diseases, Gastric pain, Gastric ulcer, Gastritis, Headache, Hemorrhoid, Hemostatic, Hepatic diseases, Hoarseness, Incontinence, Inflammatory diseases, Itching, Kidney diseases, Kidney stones, Lung disease, Mouth sores, Pilonidal sinus, Prickly heat, Prostatic cancer, Rheumatism, Sedative, Shortness of breath, Skin inflammation, Stomachache, Throat diseases, Toothache, Tranquilizer, Tuberculosis, Urinary diseases, Urinary inflammation, Vaginal discharge, Wound, Wound healing	Leaves	[15,18,19,23,36,42,44,45,46,47,50,52,53,54,57,58,60,61,63,64,68,69,72,73]
	Antipyretic, Arteriolosclerosis, Bronchitis, Cardiovascular diseases, Cough, Diabetes, Digestive, Expectorant, Gastric diseases, Gastric pain, Gastric ulcer, Gastritis, Hemorrhoid, Hemostatic, Hepatic diseases, Laxative, Respiratory diseases, Sedative, Shortness of breath, Stomachache, Throat pain, Throat diseases, Tonsillitis, Tuberculosis	Seeds	[44,69]
*Polygonum patulum* (Polygonaceae)- TK 1027	None	None	None
*Portulaca oleracea* (Portulacaceae)- TK 1048	Anemia, Anaphrodisiac, Anorexia, Anti hypertension, Anthelmintic, Appetite stimulation, Cancer, Cholesterol lowering, Common cold, Costiveness, Diabetes, Digestive, Diuretic, Fracture healing, Gastric pain, Heat rash, Heatstroke, Hemorrhoid, High fever, Hypercholesterolemia, Infertility, Inflamed wound, Intestinal diseases, Intestinal inflammation, Kidney inflammation, Kidney stone, Kidney stones, Laxative, Memory improvement, Skin inflammation, Slimming down, Sunstroke, Tranquilizer, Urinary diseases, Urinary inflammation, Uterine diseases, Wart, Wound healing, Worms,	Aerial part	[15,18,22,42,44,46,48,51,54,75]
	Anemia, Anti hypertension, Cardiac disorder, Constipation, Diabetes, Hemorrhoid, Hypercholesterolemia,	Leaves	[20,44,53]
	Digestive, Gastric pain, Infertility, Intestinal diseases, Intestinal inflammation, Laxative, Slimming down, Uterine diseases, Worms,	Seed	[44]
*Prunus armeniaca* (Rosaceae)- TK 1227	Rheumatism,	Gum	[44]
	Aperitif, Cancer, Carminative, Costiveness, Diuretic, Digestive, Intestinal spasm, Jaundice, Laxative, Worms	Fruit	[14,15,44,46,48]
	Prostatic diseases,	Leaves	[44]
	Aperitif, Bruises, Cancer, Dark circles under eye, Diabetes, Digestive, Headache, Jaundice, Laxative, Malaria, Worms	Seed	[15,44]
	Antiedemic, Bruising, Carminative, Dark circles under eye, Diuretic, Laxative,	Seed oil	[44,46,50]
*P. domestica* (Rosaceae)- TK 1218	Cold, Diabetes, Gastric pain, Headache, Hypercholesterolemia, Kidney stone, Laxative	Fruit	[15,44,55,63,76]
*P. dulcis* (Rosaceae)- TK 1065	Abdominal pain, Arteriolosclerosis, Cardiac diseases, Cough, Cuts, Diabetes, Expectoration, Expectorant, Hair care, Hair loss, High cholesterol, Hoarseness, Hypercholesterolemia, Jaundice, Kidney diseases, Kidney stone, Laxative, Rheumatism, Skin diseases, Soft care, Stress, Tonsillitis, Urinary diseases, Urinary inflammation, Wound, Wound healing, Worms	Seed	[18,19,20,26,36,44,48,49,52,54,66,67,70]
	Cough, Expectorant, Hair care, Hair loss, Headache, Hoarseness, Laxative, Rheumatism, Skin diseases, Soft care, Tonsillitis, Wound healing	Seed oil	[22,44,46]
*P. hippophaeoides* (Rosaceae)- TK 1094	Diarrhea,	Fruit	[44]
*P. microcarpa* (Rosaceae)- TK 1095	Diuretic, Kidney stone,	Cortex	[44]
	Cough, Diabetes, Diuretic, Kidney stone, Menstrual pain, Prostate, Prostatic diseases, Strengthening	Fruit	[44,64]
*P. persica* (Rosaceae)- TK 1066	Abdominal pain, Analgesic, Anthelmintic, Cough, Diabetes, Diarrhea, Earache, Eczema, Hemorrhoid, Hemostatic, Itching, Parotitis, Scorpion bite, Worms	Leaves	[42,44,46,50,52,55,60]
	Diabetes, Hemostatic, Hemorrhoid,	Seed	[44]
*Pulicaria dysenterica* subsp. *dysenterica* (Asteraceae)- TK 1055	Skin inflammation,	Capitulum	[44]
	Skin inflammation,	Leaves	[44]
*Quercus infectoria* subsp. *infectoria* (Fagaceae)- TK 1243	Stomach, Toothache,	Oak gall	[15,22],
*Rosa canina* (Rosaceae)- TK 1017	Accelerate blood circulation, Anemia, Anti hypertension, Anthelmintic, Antitussive, Angina, Arteriolosclerosis, Asthma, Bronchitis, Cancer, Cardiac diseases, Cold, Colds-Flu, Cough, Cystitis, Diabetes, Diabetes disease, Diuretic, Eczema, Embolism, Expectorant, Flu, Heart diseases, Hemorrhoid, Hemorrhoids, Hypercholesterolemia, Kidney diseases, Kidney inflammation, Kidney stone, Kidney stones, Kidney trouble, Lung pain, Malaria, Nephritis, Peptic ulcer, Pneumonia, Rash, Respiratory diseases, Shortness of breath, Thalassemia, Throat pain, Tension regulation, Tonsillitis, Tuberculosis, Urinary diseases, Urinary inflammation, Swelling,	Flowers	[15,18,23,42,44,46,52,54,56,69,71]
	Abscess, Accelerate blood circulation, Abdominal pain, Abdominal ptosis, Anemia, Anti-Allergic, Anthelmintic, Antihypertensive, Antiseptic, Antitussive, Aphrodisiac, Appetizer, Arteriolosclerosis, Asthma, Bronchitis, Cancer, Cardiac diseases, Cardiotonic and vasodilator, Carminative, Chest pain, Choler diseases, Cold, Colds and flu, Colon bleeding, Cough, Cystitis, Diabetes, Diabetes mellitus, Diarrhea, Diuretic, Digestive, Embolism, Eczema, Expectorant, Eyestrain, Flu, Gallbladder diseases, Gallstones, Gastric diseases, Gastric pain, Gastric ulcer, Gastritis, Heart diseases, Hemorrhoid, Hemorrhoids, Hepatitis, Hepatolith, Hypercholesterolemia, Hypoglycemic, Immunostimulant, Immunotonic, Infertility, Internal diseases, Intestinal carminative, Intestinal pain, Jaundice, Kidney diseases, Kidney inflammation, Kidney problems, Kidney stone, Kidney stones, Laxative, Lose weight, Lung pain, Malaria, Mycosis, Nephritis, Pass kidney stone, Peptic ulcer, Pneumonia, Psoriasis, Rash, Respiratory diseases, Respiratory tract diseases, Rheumatism, Shortness of breath, Skin diseases, Slimming down, Stomachache, Stomachache, Stomach disorders and ulcers, Stomach ulcer, Strengthening, Thalassemia, Throat pain, Tinea pedis, Tonsillitis, Tuberculosis, Tension regulation, Urinary diseases, Urinary inflammation, Wart, Wound healing, Woman sterility, Wound	Fruits	[14,18,19,20,21,22,23,26,36,42,43,44,45,46,47,48,49,50,52,53,54,55,56,57,60,61,62,63,64,67,68,69,71,72,73,75]
	Abscess, Accelerate blood circulation, Abdominal pain, Anemia, Anti-Allergic, Anti hypertension, Aperitif, Angina, Asthma, Bronchitis, Burn, Cancer, Cardiac diseases, Cardiotonic and vasodilator, Choler diseases, Colon bleeding, Cold, Cough, Cystitis, Diabetes, Diarrhea, Diuretic, Digestive, Eczema, Expectorant, Flu, Gallbladder diseases, Gallstones, Gastric diseases, Gastric pain, Gastric ulcer, Gastritis, Headache, Hemorrhoid, Hemorrhoids, Hepatolith, Hypercholesterolemia, Itching, Intestinal carminative, Intestinal pain, Jaundice, Kidney diseases, Kidney inflammation, Kidney stone, Laxative, Lung pain, Malaria, Nephritis, Pneumonia, Psoriasis, Respiratory diseases, Respiratory tract diseases, Shortness of breath, Skin diseases, Thalassemia, Throat pain, Tinea pedis, Tonsillitis, Tuberculosis, Urinary diseases, Urinary inflammation, Wart, Wound healing	Leaves	[42,44,48,52,56]
	Abscess, Anti-Allergic, Asthma, Bronchitis, Cancer, Cardiac diseases, Cardiotonic and vasodilator, Cough, Diabetes, Eczema, Expectorant, Hemorrhoid, Hemorrhoids, Itching, Peptic ulcer, Psoriasis, Rash, Respiratory tract diseases, Rheumatism, Skin diseases, Tinea pedis, Wart, Wound healing	Stipes	[42,44,48,50,57,69]
	Accelerate blood circulation, Abdominal pain, Acne, Animal treatment, Anemia, Angina, Anti hypertension, Aphrodisiac, Aperitif, Arteriolosclerosis, Bronchitis, Cancer, Cardiac diseases, Choler diseases, Cold, Colds, Colon bleeding, Cough, Diabetes, Diarrhea, Diuretic, Digestive, Expectorant, Flu, Gallstones, Gastric diseases, Gastric pain, Gastric ulcer, Gastritis, Headache, Hemorrhoid, Hemorrhoids, Hepatolith, Hypercholesterolemia, Infertility, Intestinal carminative, Intestinal pain, Jaundice, Laxative, Lung pain, Lung diseases, Malaria, Pneumonia, Rheumatism, Respiratory diseases, Shortness of breath, Swelling, Thalassemia, Throat pain, Tonsillitis, Tuberculosis, Tension regulation,	Roots	[14,23,44,50,56,57,66]
*Salvia euphratica* var. *euphratica* (Lamiaceae)- TK 1012	None	None	None
*S. multicaulis* (Lamiaceae)- TK 1161	Abdominal pain, Aperitif, Carminative, Cold, Diabetes, Digestive, Expectorant, Flu, Gastric pain, Respiratory diseases, Scorpion and snake bites, Sedative, Tonsillitis, Urinary diseases,	Aerial part	[18,20,44,45]
	Abdominal pain, Aperitif, Carminative, Cold, Digestive, Expectorant, Flu, Gastric pain, Respiratory diseases, Tonsillitis	Flower	[15,44]
	Abdominal pain, Aperitif, Burn, Carminative, Cold, Digestive, Expectorant, Flu, Gastric pain, Respiratory diseases, Swelling, Tonsillitis, Wound healing	Leaves	[15,22,44,74]
*S. palaestina* (Lamiaceae)- TK 1050	Cold, Cough, Diabetes, Expectorant, Hemostatic	Aerial part	[44]
	Wound healing,	Leaves	[44]
*Sambucus nigra* (Adoxaceae)- TK 1019	Diabetes, Diuretic, Infertility, Prostatic diseases, Prostatic Hypertrophy, Rheumatism, Swelling, Wounds (For Animals)	Areal part	[44,69]
	Wounds,	Barks	[52]
	Abscess, Antipyretic, Diaphoretic, Diabetes, Diuretic, Eczema, Emesis, Flu, Gastric pain, Laxative, Lose weight, Nausea, Rash, Skin inflammation, Sunburn, Wound healing, Wound healing –animal, Worms	Cortex	[23,44]
	Abdominal pain, Abscess, Anthelmintic, Antipyretic, Antitussive, Asthma, Bronchitis, Cold, Common cold, Cough, Diabetes, Diaphoretic, Dizziness, Diuretic, Eczema, Emesis, Expectorant, Flu, Gastric pain, Hemorrhoid, Infertility, Laxative, Lose weight, Myalgia, Nausea, Paralysis, Pinkeye, Prostatic diseases, Prostatic hypertrophy, Rash, Rheumatism, Shortness of breath, Skin inflammation, Stomachache, Sunburn, Swelling, Throat diseases, Tranquilizer, Vertigo, Whooping cough, Wound healing, Wounds (For Animals), Worms	Flowers	[23,26,36,44,46,52,59,65,69,71,73,75]
	Antipyretic, Abscess, Diabetes, Diaphoretic, Diuretic, Eczema, Flu, Hemorrhoid, Infertility, Laxative, Lose weight, Nephralgia, Prostatic diseases, Prostatitis, Rash, Skin inflammation, Sunburn, Urinary tract infection, Wound healing,	Fruits	[23,44,48,52]
	Rheumatic Pain	Herbs	[42],
	Abscess, Antipyretic, Diabetic, Diaphoretic, Diuretic, Eczema, Emesis, Flu, Gastric pain, Infertility, Laxative, Lose weight, Maturation of abscess, Myalgia, Nappy rash-baby, Nausea, Paralysis, Prostatic diseases, Prostatic hypertrophy, Prostatitis, Rash, Rheumatism, Skin inflammation, Sunburn, Swelling, Tranquilizer, Vertigo, Wound healing, Wound healing –animal, Worms	Leaves	[23,36,44,56,57,69]
	Hemorrhoids,	Seeds	[52]
	Diabetes, Diuretic, Myalgia, Paralysis, Prostatic Hypertrophy, Rheumatism, Swelling, Wounds (For Animals)	Roots	[44,69]
*Satureja hortensis* (Lamiaceae)- TK 1021	Antihypertensive, Aperitif, Cardiac diseases, Cold, Colds, Flu, Gastric pain, Headache, Immunostimulant, Stomachache	Aerial part	[44,53,71,75]
	Aperitif, Gastric pain,	Flower	[44]
	Antihypertensive, Anti hypertension, Antispasmodic, Aperitif, Cardiac diseases, Cardiac disorder, Colds, Cold, Flu, Gastric pain, Urinary inflammation	Leaves	[18,26,44,54]
*Solanum tuberosum* (Solanaceae)- TK 1225	Abscess, Analgesic, Antipyretic, Bloodshot eyes, Bruising, Burn, Conjunctivitis, Diarrhea, Edema, Eczema, Eye pain, Gastric diseases, Gastric ulcer, Headache, Pain in eyes, Pinkeye, Shortness of breath, Stomach cancer	Tubers	[44,50,52]
*Syzygium aromaticum* (Myrtaceae)- TK 1239	Mouth odor, Mouth sores, Toothache	Buds	[46]
*Teucrium polium* subsp. *polium* (Lamiaceae)- TK 1045	Abdominal ailments, Abdominal pain, Abdominal pain-baby, Ablactation, Analgesic, Aperitif, Arthralgia, Birth pains, Boil (on hand), Bronchitis, Cancer, Carminative, Carminative-baby, Cholagogic, Cold, Common cold, Cootie, Cough, Diabetes, Diabetes diseases, Diarrhea, Diuretic, Eczema, Emanagog, Epilepsy, Female diseases, Fever lowering, Flu, Headache, Hemorrhoid, Hemorrhoids, High fever, Indigestion, Infertility, Internal diseases, Kidney stone, Labor pain, Lung diseases, Lung inflammation, Malaria, Menstrual pain, Paralysis, Pneumonia, Respiratory diseases, Rheumatic pain, Rheumatism, Sedative, Shortness of breath, Skin care, Skin care-baby, Skin inflammation, Slimming down, Stimulant, Stomach diseases, Stomach ulcer, Stomachache, Strengthening, Sun stroke, Sunstroke, Sunstroke (on child), Swelling, Throat diseases, Tonic, Toothache, Tranquilizer, Tuberculosis, Urinary inflammation	Aerial part	[14,19,21,23,24,36,42,43,44,45,50,52,55,56,57,59,63,64,66,68,71,72,73,75,76]
	Abdominal pain-baby, Ablactation, Antipyretic, Antipyretic-baby, Appetizing, Bronchitis, Carminative-baby, Cholagogic, Cold, Common cold, Cough, Diabetes disease, Diuretic, Epilepsy, Flu, Hemorrhoid, Lung diseases, Malaria, Respiratory diseases, Shortness of breath, Skin care-baby, Stomachache, Throat diseases, Tuberculosis, Vertigo	Flower	[20,22,23,26,44]
	Antihypertensive, Diabetes, Diarrhea, Stomachache, Wound healing –animal,	Latex	[15,44]
	Analgesic, Antihypertensive, Antipyretic, Aperitif, Bronchitis, Carminative, Cholagogic, Cold, Common cold, Coronary failure, Cough, Diabetes, Diarrhea, Diuretic, Epilepsy, Flu, Headache, Hemorrhoid, Kidney stone, Lung diseases, Malaria, Respiratory diseases, Shortness of breath, Stomachache, Strengthening, Swelling, Throat diseases, Toothache, Tuberculosis, Urinary inflammation, Vertigo	Leaves	[18,20,23,26,44,53,66]
*Thymus kotschyanus* (Lamiaceae)- TK 1022	Abdominal ailments, Abdominal pain, Antiseptic, Aperitif, Cancer, Chest laxative, Cold, Diabetes, Diuretic, Flu, Gastritis, Hypercholesterolemia, Intestinal carminative, Sedative, Shortness of breath, Tranquilizer, Worms		[14,22,44,53,64]
	Cancer, Chest laxative, Cold, Cough, Diabetes, Hypercholesterolemia, Influenza, Shortness of breath	Leaves	[26,44]
*T. migricus* (Lamiaceae)- TK 1208	Cold, Female diseases, Menstrual pain, Shortness of breath, Throat pain		[44]
	Cold, Kidney stone,	Leaves	[75]
*Tragopogon buphthalmoides* (Asteraceae)- TK 1176	Stomachic, Wart, Wound healing,	Latex	[44,56]
	Kidney stone, Rheumatism, Stomachic, Wart,	Leaf	[44,56]
*T. dubius* subsp. *dubius* (Asteraceae)- TK 1010	Gastrointestinal disorders, Gastric pain, Intestinal diseases,	Aerial part	[21,44]
*Tribulus terrestris* var. *terrestris* (Zygophyllaceae)- TK 1230	Aphrodisiac, Arteriosclerosis, Asthma, Athlete’s foot, Cholesterosis, Diarrhea, Digestive, Diuretic, Eczema, Embolism, Gall Stones, Gastric diseases, Gastric pain, Heart regulation, Hemorrhoids, Hepatolith, High cholesterol, Jaundice, Kidney and gall Stones, Kidney ailment, Kidney diseases, Kidney inflammation, Kidney Stones, Prostate disorders, Shortness of breath, Stomachache, Urinary inflammation, Urinary tract diseases, Wart treatment	Aerial part	[18,19,21,22,23,36,44,46,48,49,52,56,64,67,72,76]
	Arteriosclerosis, Asthma, Diuretic, Expectorant, High cholesterol, Kidney ailment, Shortness of breath, Stomachache	Flower	[44,67],
	Aphrodisiac, Arteriosclerosis, Asthma, Cardiac disorder, Chest laxative, Diarrhea, Digestive, Diuretic, Gastric diseases, Gastric pain, Hepatolith, High cholesterol, Jaundice, Kidney ailment, Kidney diseases, Kidney inflammation, Kidney stone, Kidney Stones, Shortness of breath, Stomachache, Urinary inflammation, Urinary tract diseases	Fruit	[15,26,44,49,53,67,76]
	Asthma, Cardiac disorder, Chest laxative, Diarrhea, Digestive, Diuretic, Gastric diseases, Gastric pain, Hepatolith, Jaundice, Kidney diseases, Kidney inflammation, Kidney stones, Shortness of breath, Urinary inflammation	Leaves	[15,44,53]
	Kidney stone	Seed	[42]
	Atherosclerosis, Diarrhea, Digestive, Gastric diseases, Gastric pain, Hepatolith, Jaundice	Root	[44,62]
*Tripleurospermum oreades* (Asteraceae)- TK 1121	Eczema, Hair loss, Rheumatism, Wound healing,	Aerial part	[24,44]
	Eczema, Gastric pain, Hair loss, Shortness of breath, Tranquilizer, Urinary diseases, Wound healing	Capitulum	[24,44]
*Triticum* aestivum (Poaceae)- TK 1234	Analgesic,	Bran	[52]
	Anemia, Bruising, Eczema, Infertility (female), Injuries, Sprains, Sore throat, Throat pain, Wart	Seed	[44,47,50,55]
*Urtica dioica* subsp. *dioica* (Urticaceae)- TK 1131	Abdominal pain, Abscess, Acne, Anaemia, Analgesic, Anti-Allergic, Anti-dermatitis, Antihelmintic, Antifungal, Antihypertensive, Aperitif, Aphrodisiac, Arteriosclerosis, Arthritis, Arthralgia, Asthma, Baldness, Bowel, Breast cancer, Bronchitis, Cancer, Cardiac diseases, Carminative, Colds and flu, Cough, Cystitis, Depression, Dermatitis, Diabetes, Digestive, Diuretic, Emmenagogue, Enteritis, Eczema, Expectorant, Female diseases, Flu, Fracture, Gall bladder stones and sands, Gastric diseases, Gastric pain, Gastric ulcer, Gastrointestinal cancer, Gastrointestinal diseases, Genital disorders, Goiter, Hair care, Hair loss, Headache, Hemorrhage, Hemorrhoid, Hemorrhoids, Hemostatic, Hepatic diseases, Hepatitis, Hernial disk, Hormone balancer, Hypertension, Hypercholesterolemia, Hypoglycemic, Incontinence, Inflammation, Inflammatory female diseases, Infertility, Inflamed wound, Intestinal ailments, Intestinal cancer, Intestinal diseases, Intestinal inflammation, Itching, Jaundice, Joint pain, Kidney ache, Kidney diseases, Kidney inflammation, Kidney stone, Kidney stones, Larynx cancer, Laxative, Leukemia, Lipsotrichia, Liver insufficiency, Lumbago, Lung cancer, Lungs diseases, Menstrual pain, Menstrual regulator, My relaxation, Nausea, Nephritis, Nocturia, Pain, Paralysis, Prostatic cancer, Prostatic diseases, Prostatic hyperplasia, Prostatitis, Psoriasis, Pruritus, Respiratory diseases, Rheumatism, Sciatica, Shortness of breath, Skin diseases, Skin inflammation, Slimming down, Snake bite, Sterility, Stomach cancer, Stomach disorders, Stomachache, Stop bleeding, Strengthening, Throat diseases, Tinea pedis, Tonic, Toothache, Tonsillitis, Urinary diseases, Urinary disorders, Urinary inflammation, Upper respiratory tract diseases, Varicosity, Waist pain, Weight loss, Women’s diseases, Worms, Wound healing	Aerial part	[14,15,19,21,23,24,36,43,44,48,50,51,52,53,54,55,56,57,59,61,62,63,64,66,67,68,71,73,75,76]
	Abdominal pain, Abscess, Acne, Analgesic, Anti-Allergic, Anticoagulant, Antiemetic, Antifungal, Aperitif, Aphrodisiac, Arteriosclerosis, Arthralgia, Asthma, Baldness, Breast cancer, Bronchitis, Cancer, Cardiac diseases, Carminative, Cystitis, Cold and flu, Constipation, Cough, Depression, Diabetes, Diuretic, Digestive, Eczema, Edema, Emmenagogue, Expectorant, Female diseases, Flu, Fracture, Gall bladder stones and sands, Gastric diseases, Gastric pain, Gastric ulcer, Gastrointestinal cancer, Goiter, Gynecological diseases, Gynecological inflammation, Hair care, Hair loss, Headache, Hemorrhage, Hemorrhoid, Hemorrhoids, Hemostatic, Hepatic diseases, Hepatitis, Incontinence, Inflammation, Inflamed wound, Infertility, Inflammatory female diseases, Intestinal cancer, Intestinal diseases, Intestinal inflammation, Itching, Jaundice, Joint pain, Kidney ache, Kidney diseases, Kidney inflammation, Kidney stone, Larynx cancer, Laxative, Leukemia, Lumbago, Lung cancer, Lung diseases, Lungs diseases, Menstrual pain, Menstrual regulator, Menopausal symptoms, My relaxation, Nausea, Obesity, Pain, Paralysis, Prophylactic, Prostatic cancer, Prostatic diseases, Prostatic hyperplasia, Psoriasis, Relaxation, Respiratory diseases, Rheumatism, Sciatica, Shortness of breath, Skin diseases, Skin inflammation, Sterility, Stomach cancer, Stomachache, Strengthening, Throat diseases, Tinea pedis, Tonic, Toothache, Ulcer, Upper respiratory tract diseases, Urinary diseases, Urinary inflammation, Urinary system, Urinary system diseases, Weight loss, Women’s diseases, Worms, Wound healing,	Leaves	[18,20,22,26,36,44,45,47,49,51,52,54,55,58,60,63,64,66,67,69,72,73,74,75]
	Abdominal pain, Abscess, Acne, Analgesic, Anti-Allergic, Antifungal, Aperitif, Aphrodisiac, Arteriosclerosis, Arthralgia, Asthma, Baldness, Breast cancer, Bronchitis, Cancer, Carminative, Choler diseases, Cold, Cough, Cystitis, Depression, Diabetes, Diuretic, Digestive, Eczema, Emmenagogue, Expectorant, Flu, Fracture, Gastrointestinal cancer, Gastric diseases, Gastric pain, Gastric ulcer, Genital disorders, Goiter, Gynecological diseases, Gynecological inflammation, Hair care, Hair loss, Headache, Hemorrhage, Hemorrhoids, Hepatic diseases, Hepatitis, Hernial disk, Hormone balancer, Hypercholesterolemia, Incontinence, Inflammation, Infertility, Intestinal cancer, Intestinal diseases, Intestinal inflammation, Itching, Jaundice, Joint pain, Kidney ache, Kidney diseases, Kidney inflammation, Kidney stone, Larynx cancer, Laxative, Leukemia, Lumbago, Lung cancer, Lung diseases, Menstrual pain, Menstrual regulator, Menopausal symptoms, Nausea, Obesity, Paralysis, Prostatic cancer, Prostatic diseases, Prostatic hyperplasia, Psoriasis, Relaxation, Respiratory diseases, Rheumatic pain, Rheumatism, Sciatica, Shortness of breath, Skin diseases, Skin inflammation, Stomach cancer, Stomachache, Strengthening, Throat diseases, Tinea pedis, Tonic, Toothache, Upper respiratory tract diseases, Urinary diseases, Urinary inflammation, Urethral diseases, Weight loss, Wound healing, Worms	Roots	[36,44,48,50,51,52,55,60,66,67,72]
	Abdominal pain, Abortifacient, Abscess, Acne, Analgesic, Antifungal, Antihypertensive, Aperitif, Aphrodisiac, Arteriosclerosis, Arthralgia, Asthma, Breast cancer, Bronchitis, Cancer, Carminative, Choler diseases, Cold and flu, Cough, Cystitis, Depression, Diabetes, Diuretic, Digestive, Eczema, Emmenagogue, Enlarged prostate and childlessness, Expectorant, Female diseases, Flu, Fracture, Gastrointestinal cancer, Gastric diseases, Gastric pain, Gastric ulcer, Genital disorders, Goiter, Gynecological diseases, Gynecological inflammation, Hair care, Hair loss, Headache, Hemorrhage, Hemorrhoids, Hepatic diseases, Hepatitis, Hernial disk, Hormone balancer, Hypercholesterolemia, Incontinence, Inflammation, Infertility, Intestinal cancer, Intestinal diseases, Intestinal inflammation, Itching, Jaundice, Joint pain, Kidney ache, Kidney diseases, Kidney inflammation, Kidney stone, Larynx cancer, Laxative, Leukemia, Lumbago, Lung cancer, Lung diseases, Lungs diseases, Menstrual pain, Menstrual regulator, Menopausal symptoms, Nausea, Paralysis, Prostatic cancer, Prostatic diseases, Prostatic hyperplasia, Psoriasis, Relaxation, Respiratory diseases, Rheumatic pain, Rheumatism, Sciatica, Shortness of breath, Skin diseases, Skin inflammation, Stomach cancer, Stomachache, Strengthening, Throat diseases, Tinea pedis, Tonic, Toothache, Upper respiratory tract diseases, Urinary diseases, Urinary inflammation, Urinary system, Urethral diseases, Weight loss, Wound healing, Worms	Seed	[19,20,21,26,44,45,48,51,52,53,60,62,63,64,67,74]
*U. urens* (Urticaceae)- TK 1135	Abdominal pain, Abscess, Acne, Anemia, Analgesic, Antihypertensive, Arteriolosclerosis, Bronchitis, Cancer, Carminative, Cystitis, Diabetes, Diuretic, Eczema, Expectorant, Gastric diseases, Gastric pain, Gastric ulcer, Gynecological diseases, Hair loss, Headache, Hemorrhoids, Hypercholesterolemia, Hypoglycemic, Immunostimulant, Improves blood circulation, Incontinence, Infertility, Kidney diseases, Kidney inflammation, Kidney stone, Lung diseases, Menopausal symptoms, Menstrual regulator, Prostatic cancer, Prostatic diseases, Respiratory inflammation, Rheumatic pain, Rheumatism, Sciatica, Shortness of breath, Skin inflammation, Stomachache, Urethral diseases, Urinary diseases, Waist pain, Wound healing	Aerial part	[42,44,56,57,59,60,66,71,73]
	Abdominal pain, Abscess, Acne, Anemia, Analgesic, Antifungal, Antihypertensive, Asthma, Bronchitis, Cancer, Carminative, Cystitis, Diabetes, Diarrhea, Diuretic, Eczema, Expectorant, Gastric diseases, Gastric ulcer, Gynecological diseases, Hair loss, Headache, Hemorrhoids, Hypercholesterolemia, Hypoglycemic, Improves blood circulation, Incontinence, Infertility, Kidney diseases, Kidney inflammation, Kidney stone, Lung diseases, Menopausal symptoms, Menstrual regulator, Prostatic cancer, Prostatic diseases, Respiratory inflammation, Rheumatism, Shortness of breath, Skin inflammation, Stomachache, Urinary diseases, Wound healing	Leaves	[43,44,60,66,68,73,74]
	Abdominal pain, Abscess, Acne, Accelerate blood circulation, Analgesic, Anemia, Anti hypertension, Arteriolosclerosis, Bronchitis, Cancer, Diabetes, Eczema, Expectorant, Gastric pain, Hair loss, Headache, Hemorrhoids, Hypercholesterolemia, Lung’s diseases, Respiratory inflammation, Rheumatism, Shortness of breath, Skin inflammation, Wound healing	Roots	[43,44,60,66,72]
	Abdominal pain, Accelerate blood circulation, Anemia, Antifungal, Anti hypertension, Arteriolosclerosis, Asthma, Bronchitis, Cancer, Carminative, Cystitis, Diabetes, Diuretic, Expectorant, Female diseases, Gastric diseases, Gastric pain, Gastric ulcer, Hair loss, Hemorrhoids, Hypercholesterolemia, Incontinence, Infertility, Kidney diseases, Kidney inflammation, Kidney pain, Kidney stone, Lungs diseases, Menopausal diseases, Menstrual regulator, Prostatic cancer, Prostatic diseases, Respiratory inflammation, Rheumatism, Shortness of breath, Urinary diseases	Seed	[44,74]
*Verbascum georgicum* (Scrophullariaceae)- TK 1006	None	None	None
*V. sphenandroides* (Scrophullariaceae)- TK 1102	None	None	None
*Vicia hybrida* (Fabaceae)- TK 1018	None	None	None
*Viola odorata* (Violaceae)- TK 1058	Antitussive, Bronchitis, Gastric pain, Kidney pain	Aerial part	[44,46]
	Prostatic diseases,	Flower	[15,44]
	Gastric pain, Gastritis, Kidney pain, Prostatic diseases	Leaves	[15,44,64]
	Diuretic,	Root	[22]
*Vitis vinifera* (Vitaceae)- TK 1023	Abscess, Acne, Anal fistulas, Anemia, Anti-inflammatory, Antipyretic, Antipyretic in sunstroke, Bronchitis, Bruises, Burn wound care, Calcification, Cancer, Cardiovascular diseases, Cold, Common cold, Constipation, Cough, Digestive, Diuretic, Emmenagogue, Flu, Gastric diseases, Gastrointestinal diseases, Hair care, Hair loss, Hair restorer, Hemorrhoids, Hemostatic, Hypercholesterolemia, Intestinal spasm, Kidney stone, Laxative, Pain and aches, Psoriasis, Respiratory tract diseases, Rheumatic pain, Rheumatism, Skin care, Spondylitis, Strengthening, Sun stroke, Tonsillitis, Tranquilizer, Wound healing	Fruit	[15,18,36,42,44,48,50,51,53,54,66,72]
	Eye diseases,	Latex	[44]
	Abscess, Acne, Anemia, Anti-inflammatory, Antipyretic, Bronchitis, Burn wound care, Cancer, Cardiovascular diseases, Cold, Common cold, Constipation and intestinal spasm, Cough, Diaphoretic, Diuretic, Emmenagogue, Flu, Gastrointestinal diseases, Hair care, Hair loss, Hemorrhoids, Hemostatic, Hypercholesterolemia, Kidney stone, Pain and aches, Psoriasis, Respiratory tract diseases, Skin care, Tonsillitis, Wound healing	Leaves	[22,44,46,47,48]
	Anemia, Anti-inflammatory, Antipyretic, Bronchitis, Burn wound care, Cancer, Cardiovascular diseases, Constipation and intestinal spasm, Diaphoretic, Flu, Gastrointestinal diseases, Hemorrhoids, Hemostatic, Hypercholesterolemia, Respiratory tract diseases	Seed	[44,48,50]
	Abscess, Acne, Anemia, Anti-inflammatory, Antipyretic, Bronchitis, Burn wound care, Constipation and intestinal spasm, Diaphoretic, Emmenagogue, Flu, Gastrointestinal diseases, Gingivitis, Hair care, Hair loss, Inflamed gum, Psoriasis, Respiratory tract diseases, Skin care, Wound healing	Stipes	[44,46,48,57]
